# Identification of *Cis*-Regulatory Elements Involved in Mutually Exclusive Alternative Splicing of Exon 3 in *SfGluCl* from *Spodoptera frugiperda*

**DOI:** 10.3390/genes17080899

**Published:** 2026-07-30

**Authors:** Kaixiang Lin, Zijiao Song, Qiutang Huang, Ali Hasnain, Tao Tang, Wei Xu, Priscilla Amponsah, Chunqing Zhao

**Affiliations:** 1 State Key Laboratory of Agricultural and Forestry Biosecurity, College of Plant Protection, Nanjing Agricultural University, Nanjing 210095, China; linkaixiang0630@163.com (K.L.); songzijiao0505@163.com (Z.S.); hqiutang@163.com (Q.H.); alihasnain88@outlook.com (A.H.); amponsahpriscillaa@gmail.com (P.A.); 2 Key Laboratory of Integrated Pest Management on Crops in East China, Ministry of Agriculture and Rural Affair, College of Plant Protection, Nanjing Agricultural University, Nanjing 210095, China; 3 Institute of Plant Protection, Hunan Academy of Agricultural Sciences, Changsha 410125, China; tangtao@hunaas.cn; 4 School of Agricultural Sciences, Murdoch University, Perth 6150, Australia; w.xu@murdoch.edu.au

**Keywords:** *SfGluCl*, *GluCl* exon 3, mutually exclusive alternative splicing, regulatory element

## Abstract

Background/Objectives: Glutamate-gated chloride channels (GluCls) are essential inhibitory neurotransmitter receptors in insects and key targets of several insecticides. In *Spodoptera frugiperda* (FAW), mutually exclusive alternative splicing (MEAS) of exon 3 in *SfGluCl* produces three splice variants (*SfGluCl 3A*, *SfGluCl 3B* and *SfGluCl 3C*), yet the *cis*-regulatory elements involved in this splicing event remain unknown. This study aimed to identify candidate *cis*-regulatory regions associated with exon 3 MEAS and characterize their effects on exon 3 selection in a Sf9 cell-based minigene system. Methods: Semi-quantitative RT-PCR was used to assess the expression levels of three splice variants of exon 3 in *SfGluCl* across developmental stages. Comparative sequence analysis, combined with intron-deletion and exon-exchange assays, were performed to identify candidate *cis*-regulatory regions. A total of 27 constructs (23 intron-deletion and 4 exon-exchange constructs) were generated and analyzed using a minigene splicing assay in Sf9 cells. Results: Three splice variants were consistently detected across all developmental stages. In the Sf9 cell-based minigene system, two candidate *cis*-regulatory regions affecting *SfGluCl 3B* inclusion were identified: a 20 bp intronic region spanning nucleotides +13 to +32 downstream of the 5′ splice junction in the intron between exons 3B and 3C, corresponding to the region deleted in ExonB-C (Exon 3B-exon 3C)-Intdel52, and an 11 bp region within exon 3B containing six nucleotide substitutions in Ex3B (Exchanged exon 3B)-SubA2. Deleting the ExonB-C-Intdel52 or modifying the Ex3B-SubA2 significantly increased the *SfGluCl 3B* inclusion. Conclusions: These candidate intronic and exonic regions can influence exon 3B inclusion in the Sf9 cell-based minigene system. Although their functional effects and in vivo relevance remain to be validated in further study, our findings still provide a basis for future studies of *GluCl* splice variant regulation, receptor biology, and insecticide response in insects.

## 1. Introduction

The fall armyworm (FAW), *S. frugiperda* (J.E. Smith), is a major agricultural insect pest that originated in the Americas. Owing to its ability to utilize a wide range of host plants [1], FAW has spread rapidly into Africa, Asia, and Oceania in recent years [2,3], posing a serious threat to crop production worldwide. Chemical control remains a major strategy for FAW management, and several insecticides used for chemical control targeting glutamate-gated chloride channels (GluCls). GluCls belong to the cys-loop ligand-gated ion channel (LGIC) superfamily and are major inhibitory neurotransmitter receptors in insects. They structurally form pentameric complexes with each subunit comprising four domains: an N-terminal hydrophilic region containing the ligand-binding site, three transmembrane segments (TM1-TM3), a variable intracellular hydrophilic loop, and a C-terminal hydrophobic region containing the fourth transmembrane segment (TM4) [4]. Functionally, GluCls mediate fast inhibitory synaptic transmission, which is essential for neural signal transduction [5]. GluCls were first cloned in the nematode *Caenorhabditis elegans* and have subsequently been identified in a broad range of invertebrates, including insects [6]. The differences in structure and function of GluCls between insects and vertebrates make them attractive targets for the development of selective insecticides. Notably, alternative splicing (AS) is an important mechanism contributing to the functional diversity of GluCls, as splice variants can differ in receptor expression or pharmacological properties.

In eukaryotes, AS is a crucial post-transcriptional regulatory mechanism and generates multiple mRNA variants from one pre-mRNA transcript, which expands proteomic complexity [7]. To date, five major types of AS are recognized: exon skipping, intron retention, alternative 5′ splice sites, alternative 3′ splice sites, and mutually exclusive alternative splicing (MEAS) [8,9,10,11,12]. Among these, MEAS involves the selective inclusion of one exon from a cluster of adjacent alternative exons in the mature mRNA.

In insects, exon 3 of *GluCl* genes undergoes MEAS, producing three distinct splice variants: *GluCl 3A*, *GluCl 3B*, and *GluCl 3C* [13,14,15,16]. These splice variants exhibit functional divergence in neuronal signaling and contribute to insecticide resistance [17,18,19]. Heterologously expressed splice variants reveal different sensitivities to agonists and antagonists. For instance, *GluCl* splice variants in *Musca domestica* (*MdGluCl*) and *Anopheles gambiae* (*AgGluCl*) show varied responses to fipronil and picrotoxinin [18,20]. Similarly, splice variants of *Bombyx mori GluCl* (*BmGluCl*) demonstrate different maximal responses to L-glutamate and ivermectin (IVM) [17]. Moreover, *Tribolium castaneum* splice variant GluCl-3b (*TcGluCl-3b*) exhibits tissue-specific enrichment in the midgut, suggesting its special role in intestinal physiology [21]. Together, these findings indicate that MEAS of exon 3 contributes to the molecular and functional diversity of insect GluCls. Identifying the *cis*-regulatory elements, that govern exon 3 selection in FAW, is therefore important for understanding how specific *SfGluCl* splice variant profiles are generated. Because splice variants of exon 3 are different in receptor function and insecticide sensitivity of neuron receptor, identifying the *cis*-regulatory elements that regulate exon 3 MEAS will provide a basis for future studies of GluCl function and insecticide response in FAW.

However, the *cis*-regulatory elements, that regulate the *GluCl* exon 3 MEAS, have not been experimentally characterized in any insect species. Here, we analyzed exon 3 MEAS in *SfGluCl* from FAW using sequence comparison, intron-deletion analysis, and exon-exchange assays. Our minigene assays identified a 20 bp intronic region corresponding to the sequence deleted in ExonB-C-Intdel52 and an 11 bp exonic region modified in Ex3B-SubA2 as candidate *cis*-regulatory regions affecting exon 3B inclusion in the Sf9 cell-based minigene system. Here, candidate *cis*-regulatory regions refer to sequence intervals whose deletion or substitution altered exon 3 splicing in this system. These findings provide a basis for further defining the *cis*-regulatory architecture of *SfGluCl* exon 3 splicing.

## 2. Materials and Methods

### 2.1. Insect Strain

FAW was a susceptible laboratory strain originally derived from the field of Guangdong Province, China, in April 2019, and has been maintained in the laboratory for 71 generations without exposure to any insecticides. FAW was collected and reared on an artificial diet as previously described [22]. Larvae were kept in disposable plastic containers at 26 ± 2 °C, 60–70% relative humidity, and a 16:8 h (light:dark) photoperiod, with fresh artificial food replenished daily. Late sixth-instar larvae were transferred to individual disposable cups for pupation. Adults were paired at a 1:1 (♀:♂) ratio in mesh cages lined with plastic wrap to facilitate oviposition and provided with cotton balls soaked in 10% (*w*/*v*) honey solution for nutrients.

### 2.2. Bioinformatic Analysis

To identify conserved candidate *cis*-regulatory regions contaning elements, the *GluCl* intronic and exonic sequences from 24 lepidopteran species were retrieved and subjected to multiple sequence alignment (Table S1). The alignment was performed using MUSCLE as implemented in MEGA X 10.1 (https://www.megasoftware.net/, accessed on 10 November 2022) by using the default settings: a gap-opening penalty of −400.00, a gap-extension penalty of 0.00, a maximum of 16 iterations, UPGMA clustering for all iterations, and a minimum diagonal length (Lambda) of 24.

### 2.3. In Silico Prediction of RBP-Binding Motifs, Homolog Identification, and AlphaFold3-Based Protein–RNA Complex Modeling

Predicted RNA-binding protein (RBP)-binding motifs within the candidate *cis*-regulatory regions identified by the minigene assays were analyzed using RBPmap (http://rbpmap.technion.ac.il/, accessed on 3 July 2026) and the ATtRACT database (https://attract.cnic.es/, accessed on 3 July 2026). For the region corresponding to ExonB-C-Intdel52, the pre-mRNA sequence spanning the 20-nt intronic candidate *cis*-regulatory region and its flanking intronic sequences was analyzed. For the region corresponding to Ex3B-SubA2, the exon 3B sequence spanning the positions modified in the Ex3B-SubA2 construct, together with flanking exonic sequences, was analyzed. Predicted RBP-binding motifs located within or overlapping the experimentally defined candidate *cis*-regulatory regions were retained for subsequent analysis.

To determine whether corresponding RBP families are represented in FAW, reference predicted RBPs were used as BLASTP queries against the NCBI protein database (https://blast.ncbi.nlm.nih.gov/Blast.cgi, accessed on 4 July 2026) restricted to FAW. RBP homologs were assigned based on the best BLASTP hits, and the conserved domains of RBP homologs were examined using NCBI Conserved Domain Search (https://www.ncbi.nlm.nih.gov/Structure/cdd/cdd.shtml, accessed on 4 July 2026).

To further explore the structural plausibility of interactions between candidate FAW RBP homologs and the corresponding candidate *cis*-regulatory regions, the protein–RNA complex was predicted using AlphaFold3 Server (https://alphafoldserver.com/, accessed on 9 July 2026). The FAW homolog heterogeneous nuclear ribonucleoprotein A1 (SfhnRNP A1-like, XP_035441205.1, Text S1) was modeled with the pre-mRNA fragment corresponding to the region deleted in the ExonB-C-Intdel52 construct and the RBP Musashi homolog Rbp6 (SfRbp6-like, XP_035437037.1, Text S1) was modeled with the RNA fragment corresponding to the region modified in the Ex3B-SubA2 construct. Model confidence was evaluated using the interface predicted template modeling score (ipTM), with higher values indicating greater confidence in the predicted interchain arrangement.

### 2.4. Minigene Construction, Deletion, Exon Exchange, and Transfection

The minigene spanned constitutive exons 2 and 4, and the intervening genomic sequence between them. The corresponding exon 2-4 sequence derived from the genome assembly and the experimentally sequenced exon 2-4 sequence are provided in Text S2 and S3, respectively. Genomic DNA was extracted from FAW and used as a template for PCR amplification of the *SfGluCl* exon 3 minigene using a T100 Thermal Cycler (Bio-Rad Laboratories, Inc., Hercules, CA, USA). The PCR mixture contained 0.4 μL each of forward and reverse primers (0.4 μM final concentration), 5 μL of 2 × Phanta Flash Master Mix (Dye Plus, Vazyme Biotech Co., Ltd., Nanjing, Jiangsu Province, China), 0.4 μL genomic DNA (250 ng/μL), and 3.8 μL nuclease-free water. PCR conditions were as follows: initial denaturation at 98 °C for 30 s; 34 cycles of denaturation (98 °C, 10 s), annealing (60.3 °C, 5 s), and extension (72 °C, 160 s); followed by a final extension at 72 °C for 1 min. PCR products were verified via 0.8% agarose gel electrophoresis and purified using the TIANquick Midi Purification Kit (Tiangen Biotech Co., Ltd., Beijing, China).

**Figure 1:**
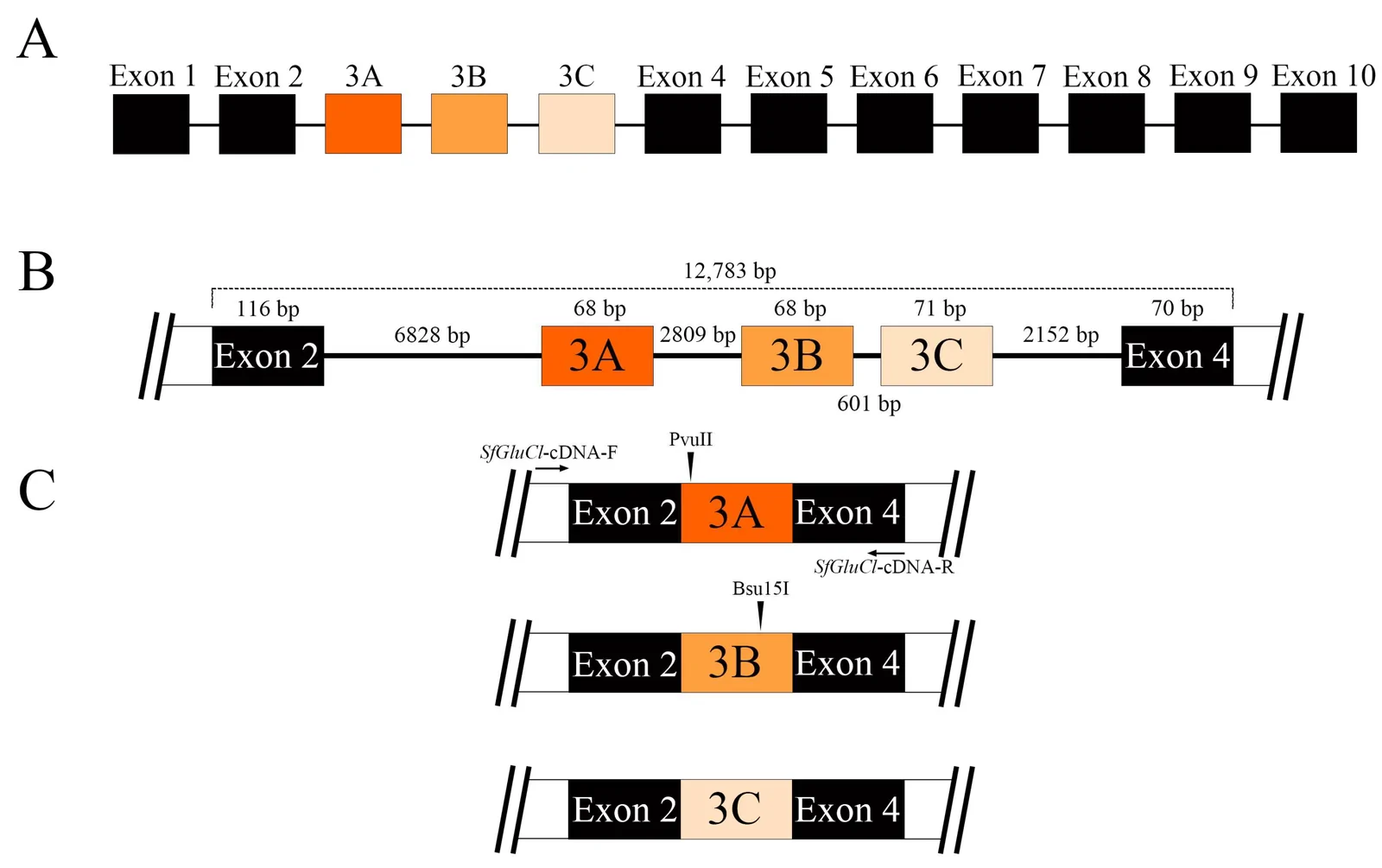
Genomic structure and mutually exclusive alternative splicing (MEAS) of *SfGluCl* exon 3. (**A**) Genomic organization of *SfGluCl*. (**B**) The exon 3 minigene was constructed to encompass the constitutive exon 2, the constitutive exon 4, and the entire intervening genomic sequence. (**C**) Schematic representation of the three MEAS variants of exon 3 amplified by RT-PCR. The expected RT-PCR products were 391 bp for *SfGluCl 3A* and *SfGluCl 3B*, and 394 bp for *SfGluCl 3C*. *SfGluCl 3A* was digested by PvuII into 262 and 129 bp fragments, and *SfGluCl 3B* was digested by Bsu15I into 305 and 86 bp fragments; *SfGluCl 3C* lacked both restriction sites and cannot be digested.

The minigene, which comprised constitutive exon 2, exon 4, and the intervening sequence between them (Figure 1B), was cloned into the pMIB/V5-His B vector (Text S4), provided by Professor Fuqiang Xu (Chinese Academy of Sciences, Beijing, China) using the Vazyme ClonExpress Ultra One Step Cloning Kit V2 (Vazyme Biotech Co., Ltd., Nanjing, Jiangsu Province, China). This vector utilizes the *Orgyia pseudotsugata* immediate-early 2 (OpIE2) promoter and contains a native OpIE2 polyadenylation sequence. Forty-eight clones carrying the wild-type (WT) constructs were initially screened and confirmed by General Biol Co., Ltd. (Chuzhou, China). Intron-deletion and exon-exchange constructs were generated through homologous recombination-based modification of the WT constructs. The WT constructs and, all intron-deletion and exon-exchange constructs used for transfection were verified by Sanger sequencing. For each construct, one sequence-verified plasmid clone was used for transfection. The constructs were transfected into Sf9 cells using Lipofectamine 3000 (Thermo Fisher Scientific, Waltham, MA, USA) as previously described [23], with three technical replicates for each construct.

### 2.5. RNA Extraction and cDNA Synthesis

Total RNA was extracted using TRIzol Reagent (Thermo Fisher Scientific, Waltham, MA, USA) from eggs, first- to sixth-instar larvae, pupae, female adults, male adults, and transfected Sf9 cells collected 48 h after transfection. RNA samples were stored at −80 °C. For insect developmental samples, pooled samples (50 mg) were prepared for RNA extraction and cDNA synthesis. For the Sf9 cell-based minigene system, the same sequence-verified plasmid construct was transfected into Sf9 cells.

Total RNA was assessed for quality by A260/280 ratio using a Nanodrop 1000 spectrophotometer (Thermo Fisher Scientific, Waltham, MA, USA) and for integrity by 1.2% agarose gel electrophoresis. cDNA was synthesized from 1 μg total RNA using the PrimeScript RT reagent Kit with gDNA Eraser (Takara Bio Inc., Kusatsu, Shiga, Japan). Reverse transcription was performed under standardized conditions: 2 μL of 8× gDNA Eraser Premix, 1 μg of total RNA, and RNase-free H_2_O were mixed to a final volume of 16 μL. The reaction program was set as follows: 42 °C for 2 min. Subsequently, 4 μL of 5× RT Premix was added, and the reaction proceeded under the following protocol: 37 °C for 10 min and 85 °C for 5 s. Synthesized cDNAs were stored at −20 °C for gene cloning.

### 2.6. Semi-Quantitative RT-PCR

To determine the optimal conditions for semi-quantitative RT-PCR, preliminary trials using different genes (Table S2) were conducted with 26, 30, 32, 34, and 36 amplification cycles (Figure S1). Amplified DNA fragments were separated on 1.5% agarose gels, and band intensities were quantified using ImageJ v1.8 (https://imagej.en.softonic.com/, accessed on 3 March 2023) based on integrated density values. PCR reactions (15 μL total volume) included 0.6 μL each of forward and reverse primers (0.4 μM final concentration for each primer), 7.5 μL of 2 × Rapid Taq Master Mix (Vazyme Biotech Co., Ltd., Nanjing, Jiangsu Province, China), 1 μL cDNA, and 5.3 μL nuclease-free water. The cycling protocol was: 95 °C for 3 min, 34 cycles of 95 °C for 15 s, 60 °C for 15 s, and 72 °C for 6 s; followed by a final extension at 72 °C for 5 min. RT-PCR assays were performed with three technical replicates for each developmental stage. The ribosomal protein L10 (*RPL10*) was used as the reference gene [24]. This semi-quantitative RT-PCR analysis was used to compare relative expression trends and splice variant inclusion patterns rather than to determine absolute transcript abundance.

### 2.7. Detection of Minigene Transfection Outcomes and Grayscale Analysis

To avoid interference from endogenous *GluCl* transcripts, a 5′-vector-specific primer (Table S2) was designed for PCR amplification using synthesized cDNA as the template. Sequence alignment using DNAMAN version 7 (Lynnon Biosoft, San Ramon, CA, USA) confirmed that this 5′-vector-specific primer does not match sequence in endogenous *SfGluCl* cDNA. Therefore, it specifically amplified the transfected constructs. Each PCR reaction mixture consisted of 2.0 μL each of forward and reverse primers (0.4 μM final concentration, Table S2), 25 μL 2 × Phanta Max Master Mix (Vazyme Biotech Co., Ltd., Nanjing, Jiangsu Province, China), 5.0 μL cDNA template (undiluted), and 16 μL nuclease-free water. The thermal cycling conditions were 95 °C for 3 min; 34 cycles of 95 °C for 15 s, 57 °C for 15 s, and 72 °C for 30 s, followed by a final extension at 72 °C for 5 min. PCR products were digested with exon-specific restriction enzymes (Thermo Fisher Scientific, Waltham, MA, USA): PvuII for *SfGluCl 3A*, and AatII for *SfGluCl 3B*. Representative undigested and restriction-enzyme-digested RT-PCR products are shown in Figure S2. Band intensities were quantified using ImageJ v1.8.

### 2.8. Statistical Analysis

PCR amplicons of *SfGluCl 3A*, *SfGluCl 3B*, and *SfGluCl 3C* from different developmental stages were analyzed by measuring the grayscale intensity (GSI) of agarose gel electrophoresis bands using ImageJ v1.8. After background subtraction, the band intensity of each target gene was normalized to that of *RPL10* [24] from the same sample, and the normalized grayscale values were used as the primary variables for subsequent statistical analyses. The sample with the lowest normalized expression level was selected as the calibrator to calculate the relative expression levels of the target genes at different developmental stages. The relative proportion of each splice variant was calculated using the following formula: *SfGluCl 3X*/(*SfGluCl 3A* + *SfGluCl 3B* + *SfGluCl 3C*) × 100%, where X represents *SfGluCl 3A*, or *SfGluCl 3B*, or *SfGluCl 3C*. Statistical analyses were performed using normalized expression levels or the proportions of splice variants. Differences among groups were assessed by one-way analysis of variance (ANOVA), followed by Tukey’s test. Differences were considered statistically significant at *p* < 0.05.

For analysis of the constructs, band intensities from three technical replicates were quantified using ImageJ v1.8. The GSI of the intact exon 2-3X-4 band in the undigested control was used as the total GSI for each construct, where 3X represents *SfGluCl 3A*, *SfGluCl 3B*, or *SfGluCl 3C*. After PvuII digestion, the remaining intact exon 2-3X-4 band represented the combined GSI of *SfGluCl 3B* and *SfGluCl 3C*. The *SfGluCl 3A* GSI was calculated by subtracting this value from the total GSI. Similarly, after Bsu15I digestion, the remaining intact band represented the combined GSI of *SfGluCl 3A* and *SfGluCl 3C*. The *SfGluCl 3B* GSI was calculated by subtracting this value from the total GSI. The *SfGluCl 3C* GSI was then inferred by subtracting the GSI of *SfGluCl 3A* and *SfGluCl 3B* from the total GSI. The inclusion level of each splice variant was calculated as the GSI of splice variant divided by the total GSI. The *SfGluCl 3C* intensity was inferred by subtraction rather than measured from a uniquely isolated diagnostic band. Therefore, uncertainties in total-band densitometry, background subtraction, and restriction digestion-based estimates of *SfGluCl 3A* and *SfGluCl 3B* may propagate into the calculated *SfGluCl 3C* value.

Statistical analysis was performed with SPSS 22.0 (SPSS Inc., Chicago, IL, USA). Data are presented as mean ± standard error of the mean (SEM) from three technical replicates for each construct. Statistical significance was assessed using a two-tailed Student’s *t*-test. Figures were generated using GraphPad Prism 6.0 (GraphPad Software Inc., La Jolla, CA, USA).

## 3. Results

### 3.1. Temporal Expression Profile of SfGluCl Splice Variants Across Developmental Stages

**Figure 2:**
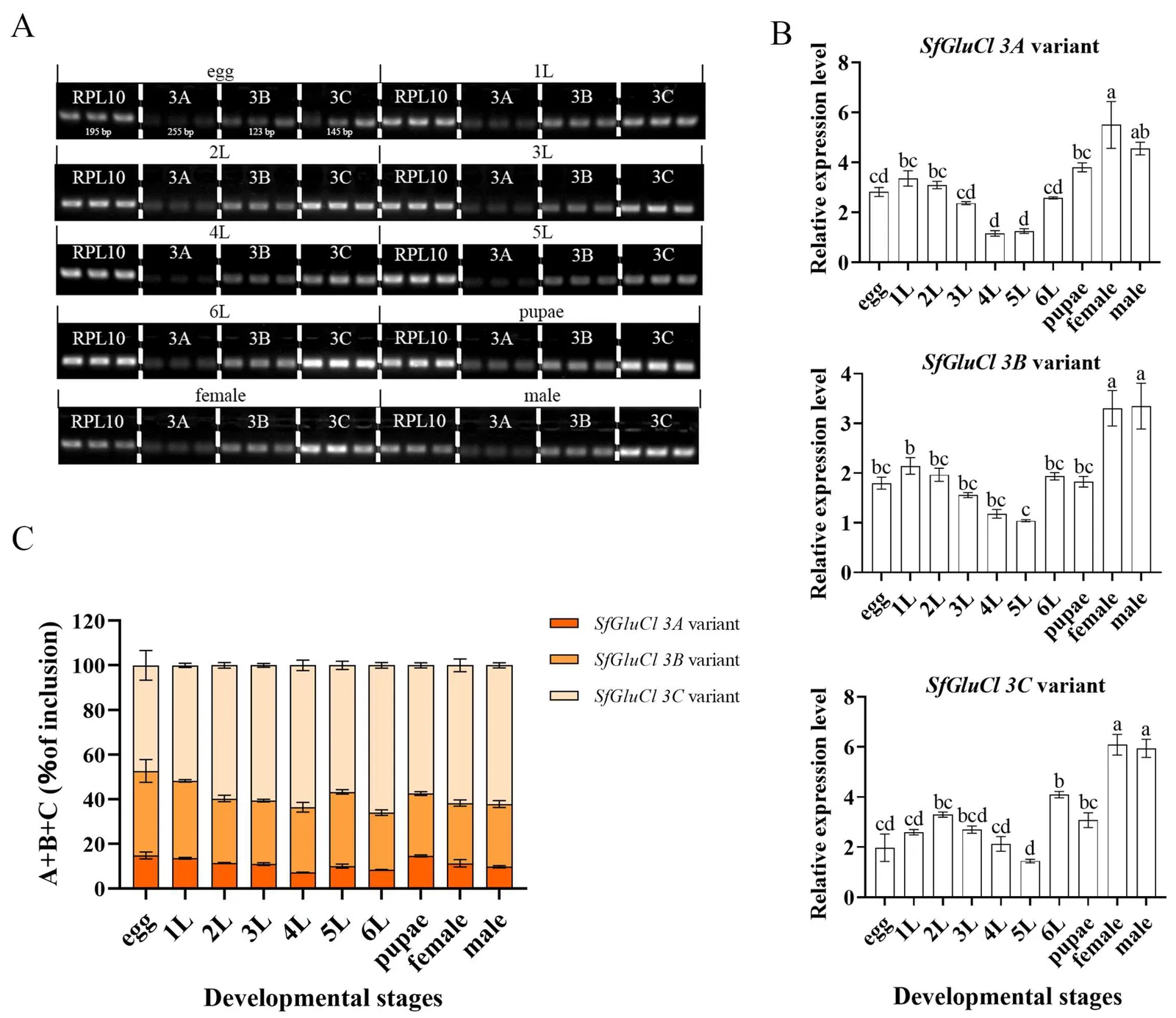
Developmental expression and relative inclusion of *SfGluCl* splice variants. (**A**) Semi-quantitative RT-PCR electrophoretogram of *SfGluCl* exon 3 splice variants. *RPL10* was used as the internal reference gene. (**B**) Relative expression levels of *SfGluCl 3A*, *SfGluCl 3B*, and *SfGluCl 3C* at egg, first- (L1), second-(L2), third (L3)-, fourth (L4)-, fifth (L5)-, and sixth (L6)-instar larvae, pupae, female adults and male adults. Relative expression levels were calculated by normalizing the band intensity of each splice variant to that of *RPL10*. (**C**) Inclusion percentages of *SfGluCl 3A*, *SfGluCl 3B*, and *SfGluCl 3C* at different developmental stages. The inclusion percentage of each splice variant was calculated as the band intensity of that splice variant divided by the combined band intensity of the *SfGluCl 3A*, *SfGluCl 3B*, and *SfGluCl 3C*. Data were obtained from three technical replicates for each developmental stage. Statistical significance was assessed using one-way ANOVA followed by Tukey’s test. Error bars represent the standard error of the mean (SEM). Significance is shown by different lowercase letters (*p* < 0.05).

The relative expression levels of *SfGluCl 3A*, *SfGluCl 3B*, and *SfGluCl 3C* across different developmental stages are shown in Figure 2A,B. Using the fourth-instar larval stage as the reference, the relative expression levels of *SfGluCl 3A* in eggs, first- to third-instar larvae, fifth- and sixth-instar larvae, pupae, female adults, and male adults were 2.43-, 2.89-, 2.67-, 2.04-, 1.08-, 2.22-, 3.28-, 4.47-, and 3.93-fold higher, respectively. Relative to the fifth-instar larval stage, the expression levels of *SfGluCl 3B* in the other developmental stages ranged from 1.13- to 3.21-fold. Using the egg stage as the reference, *SfGluCl 3C* expression in the remaining developmental stages ranged from 0.73- to 3.08-fold.

Overall, the three splice variants showed broadly comparable developmental patterns in relative expression level, although the extent of the observed changes varied among them. Their relative expression levels increased from the egg stage to the first-instar larval stage, gradually declined from the second to the fifth instar, and then increased again from the sixth instar onward, with the highest levels observed in adults. Specifically, *SfGluCl 3A* showed the lowest expression level at the fourth instar larvae, whereas *SfGluCl 3B* and *SfGluCl 3C* showed the least expression level at the fifth instar larvae. In the developmental stages of FAW, *SfGluCl 3C* remained the predominant splice variant (Figure 2C). Although minor fluctuations were observed among developmental stages, the overall inclusion pattern of the three exon 3 splice variants remained relatively stable.

### 3.2. Intronic Regulation of MEAS

As the first step, to identify intronic sequences regulating the MEAS of *SfGluCl* exon 3, we first generated four intron-deletion constructs (Figure S3A): Exon2-A-Intdel1, ExonA-B-Intdel1, ExonB-C-Intdel1, and ExonC-4-Intdel1, in which the intronic regions between exon 2 and exon 3A, exon 3A and exon 3B, exon 3B and exon 3C, and exon 3C and exon 4, respectively, were deleted. After transfection into Sf9 cells, RT-PCR and GSI analysis showed that the four intron-deletion constructs altered splice variant inclusion relative to the WT construct (Figure S3B), suggesting that these introns may contain candidate *cis*-regulatory regions affecting exon 3 splice variant selection.

**Figure 3:**
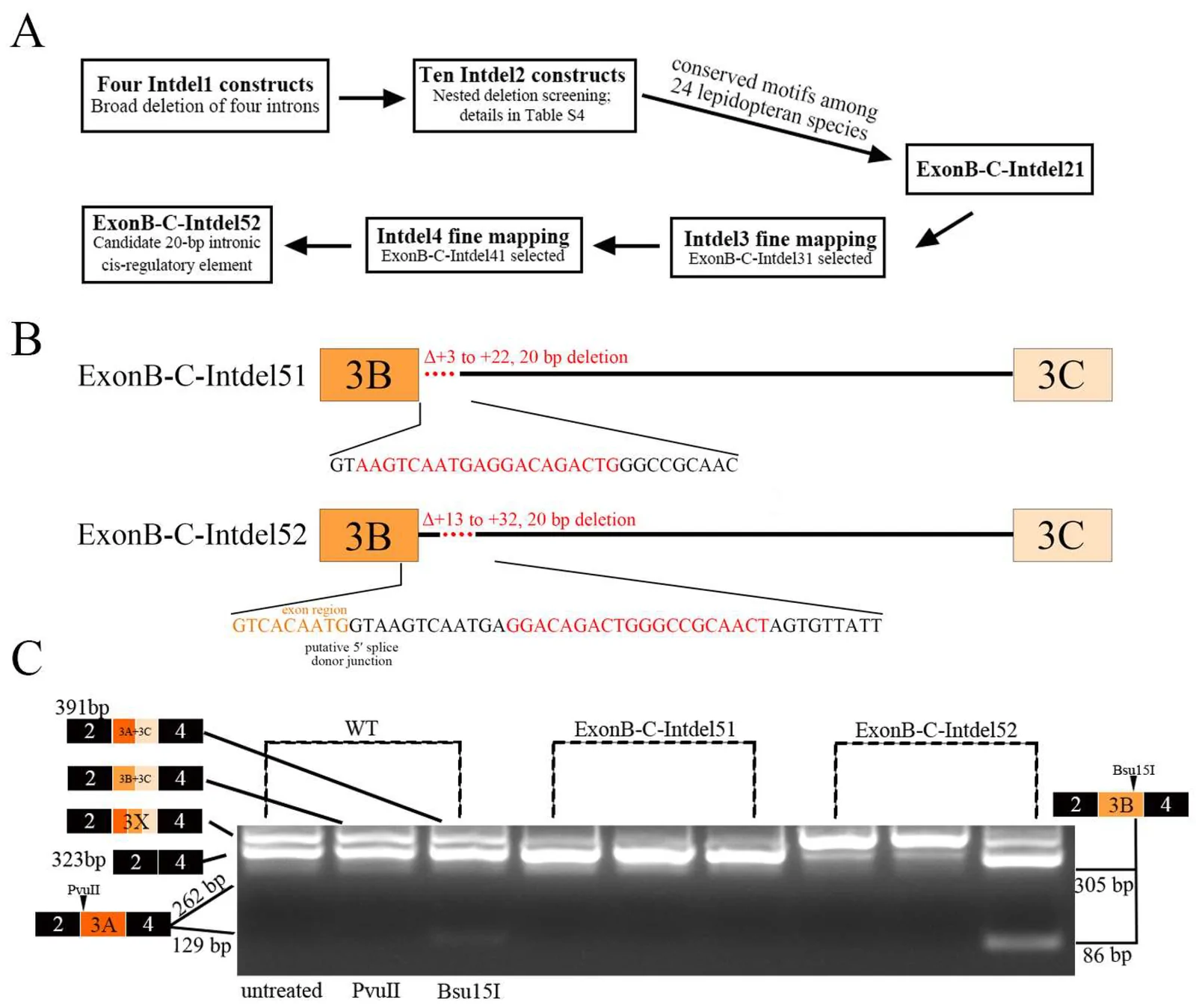
Overview of the stepwise intron-deletion strategy and analysis of the ExonB-C-Intdel51 and ExonB-C-Intdel52 constructs. (**A**) Overview of the stepwise intron-deletion strategy from the initial Intdel1 screening to identification of ExonB-C-Intdel52. (**B**) Schematic diagrams of the ExonB-C-Intdel51 and ExonB-C-Intdel52 deletion constructs. The exon 3B/intron boundary is indicated as the putative 5′ splice donor junction. Nucleotide positions are numbered relative to the first intronic nucleotide downstream of exon 3B. Orange and light-orange boxes indicate exon 3B and exon 3C, respectively. Red nucleotides and red dashed lines indicate the deleted intronic sequences. ExonB-C-Intdel51 deletes nucleotides +3 to +22, whereas ExonB-C-Intdel52 deletes nucleotides +13 to +32, leaving the first 12 intronic nucleotides downstream of exon 3B intact. (**C**) RT-PCR assays of the ExonB-C-Intdel51 and ExonB-C-Intdel52 constructs. Red, orange and light-orange boxes indicate the exon 3 splice variants, whereas black boxes indicate the constitutive exons 2 and 4.

As the second step, a series of nested deletions was designed for the four intron-deletions in the Intdel1 constructs (Figure S3), including three deletions in Exon2-A-Intdel1, and ExonA-B-Intdel1, two deletions in ExonB-C-Intdel1 and ExonC-4-Intdel1, respectively (Figure S4A,B). Based on the splice variant changes observed relative to the WT construct, Exon2-A-Intdel21, ExonA-B-Intdel21, ExonB-C-Intdel21, and ExonC-4-Intdel21 were selected as representative candidate *cis*-regulatory regions for subsequent comparison. As summarized in Figure 3A and Table S3, four Intdel1 constructs were subjected to nested Intdel2 screening; ExonB-C-Intdel21 was then prioritized based on conserved sequences, and successive Intdel3–Intdel5 deletions progressively narrowed the candidate *cis*-regulatory region to ExonB-C-Intdel52.

As the third step, to select one intron for further deletion analysis, we integrated the deletion results with comparative sequence analysis across 24 lepidopteran species (Table S1). Among the four introns, the intron between exons 3B and 3C was prioritized because a cluster of highly conserved sequences was identified in this intron (Figure S5). Therefore, ExonB-C-Intdel21 was further subdivided into four additional deletions: ExonB-C-Intdel31, ExonB-C-Intdel32, ExonB-C-Intdel33, and ExonB-C-Intdel34 (Figure S6A; Table S3). Deleting ExonB-C-Intdel31 reduced the inclusion of *SfGluCl 3B* to 3.88% (−34.43%) and increased that of *SfGluCl 3A* to 34.51% (+26.21%), suggesting that this region is important for *SfGluCl 3B* splicing. No significant change was observed for the other three deletions (Figure S6B).

As the fourth step, three additional nested deletions, ExonB-C-Intdel41, ExonB-C-Intdel42, and ExonB-C-Intdel43, were generated within the ExonB-C-Intdel31 interval (Figure S7A; Table S3). Deleting the ExonB-C-Intdel41 region reduced the inclusion of *SfGluCl 3B* to 1.25% (−37.06%) and increased that of *SfGluCl 3C* by 33.87%, indicating that this fragment may represent a candidate *cis*-regulatory region for *SfGluCl 3B*. No significant change was detected for the other two deletions (Figure S7B).

**Figure 4:**
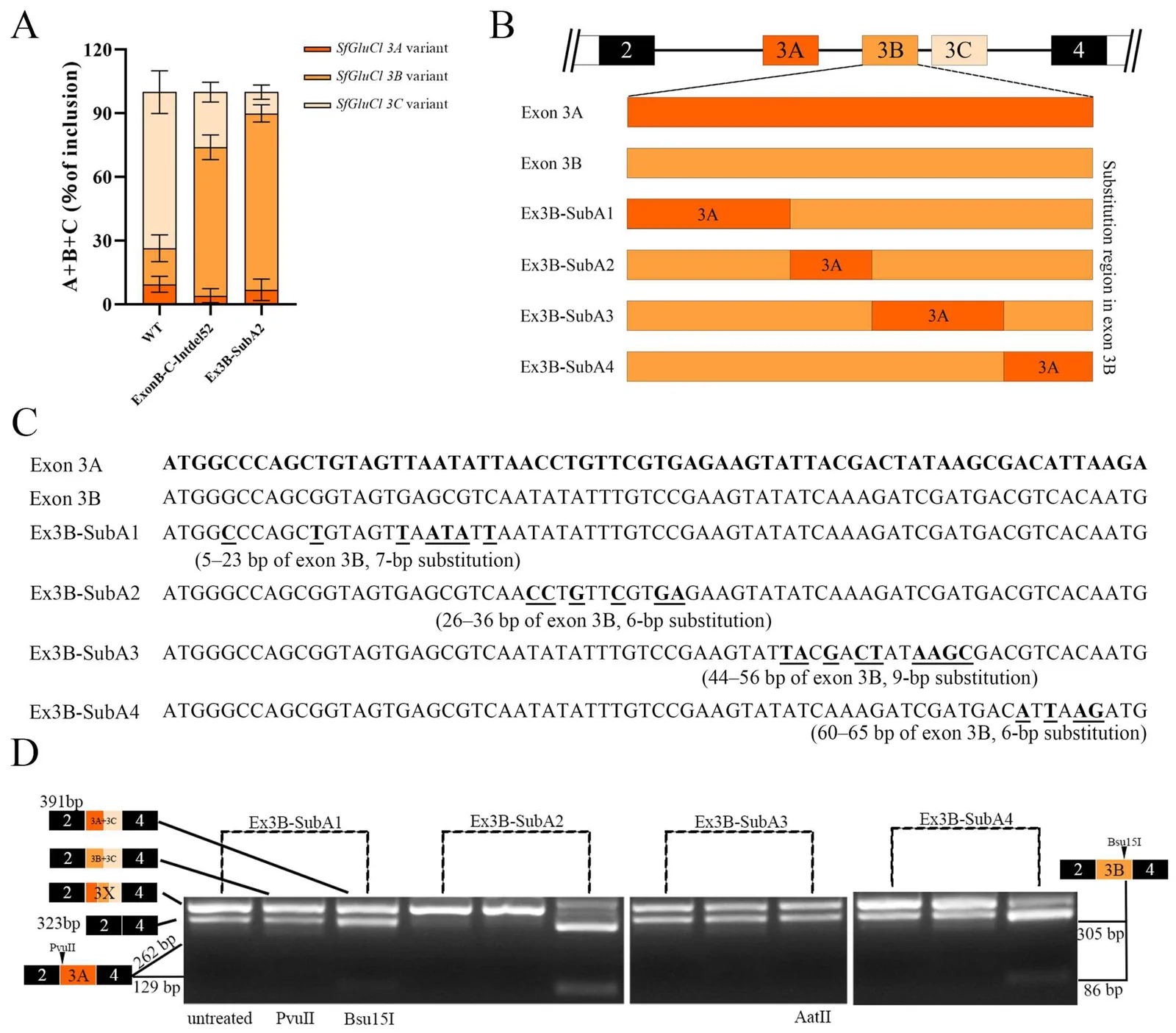
Exon-exchange analysis of *SfGluCl* exon 3 splicing. (**A**) Inclusion of *SfGluCl 3A*, *SfGluCl 3B*, and *SfGluCl 3C* in the constructs. Error bars represent the SEM. (**B**) Schematic diagrams of exon-exchange constructs in which short regions of exon 3B were replaced with the corresponding sequences from exon 3A. (**C**) Sequence map of the exon-exchange regions. Nucleotide positions are numbered relative to the first nucleotide of exon 3B, and substituted nucleotides are shown in bold and underlined. (**D**) RT-PCR and restriction-enzyme digestion assays of the exon-exchange constructs.

As the final step, two additional deletions, ExonB-C-Intdel51 and ExonB-C-Intdel52, were introduced within the ExonB-C-Intdel41 interval for further deletion analysis (Figure 3B; Table S3). It is well-established that the highly conserved core of a typical eukaryotic 5′ splice donor site extends approximately 6 nucleotides into the intron [25]. While the deletion in ExonB-C-Intdel51 encroaches upon this region and may affect the basal splicing machinery, the ExonB-C-Intdel52 deletion was meticulously designed to start 12 bp downstream of the 5′ splice junction. This strategic positioning safely bypasses the canonical motif, leaving the putative 5′ splice donor junction completely intact. Deleting the ExonB-C-Intdel51 region did not significantly alter splice variant selection. In contrast, deletion of the 20 bp region in the ExonB-C-Intdel52 construct significantly increased *SfGluCl 3B* inclusion from 16.96 ± 3.63% to 69.93 ± 3.32% (*p* < 0.001), and decreased *SfGluCl 3C* inclusion from 73.53 ± 5.82% to 25.95 ± 2.68% (*p* = 0.002) (Figure 3C and Figure 4A). These results suggested that the 20 bp region deleted in the ExonB-C-Intdel52 construct should contain a candidate intronic *cis*-regulatory element that influences *SfGluCl 3B* inclusion in the Sf9 cell-based minigene system.

### 3.3. Exonic Regulation of MEAS

To explore whether exonic sequences contribute to the MEAS of *SfGluCl* exon 3 splice variants, we conducted targeted exon-exchange experiments. Pairwise alignments revealed that exons 3A and 3B share the highest sequence identity (61.76%). Given this high homology, we hypothesized that the specific *cis*-regulatory elements discriminating both exons are located within their divergent segments. Therefore, we targeted a short divergent segment within exon 3B and replaced it with the homologous sequence from exon 3A to generate the constructs (Figure 4B,C), aiming to pinpoint the key regulatory element. Specifically, in the Ex3B-SubA2 construct, the exon 3B sequence at positions 26–36 (TATATTTGTCC) was replaced with the exon 3A-derived sequence CCTGTTCGTGA, involving six nucleotide substitutions within the 11 bp region (Figure 4C). In the Ex3B-SubA2 construct, the inclusion of *SfGluCl 3B* was increased from 16.96 ± 3.63% to 83.10 ± 2.35% (*p* < 0.001), while that of *SfGluCl 3C* decreased from 73.53 ± 5.82% to 10.01 ± 1.93% (*p* < 0.001) (Figure 4A), suggesting that the 11-bp exonic region modified in the Ex3B-SubA2 construct may contain a candidate exonic *cis*-regulatory element that influences exon 3 splice variant selection in the Sf9 cell-based minigene system.

### 3.4. In Silico Identification of Candidate RBPs and AlphaFold3-Based Protein–RNA Complex Prediction

To further explore potential trans-acting factors associated with the two-candidate *cis*-regulatory regions identified by the minigene assays, RBP motif was predicted by using RBPmap and ATtRACT. For ExonB-C-Intdel52, RBPmap predicted an HNRNPA1-related motif with the matched sequence ggacag, whereas ATtRACT predicted HNRNPF/H-related *ugggc* motifs within this region (Table S4). For Ex3B-SubA2, RBPmap predicted several hnRNP-family motifs fully located within the substituted sequence, including HNRNPA0, HNRNPA1, HNRNPC, and HNRNPDL motifs (Table S4). Because hnRNP-, SR/SRSF-, and SF-related proteins are commonly associated with pre-mRNA splicing, RBP families related to these were selected for subsequent homology analysis. BLASTP identified putative FAW homologs of the selected RBP families (RBP homologs), and conserved-domain analysis showed that these homologs contained RNA-recognition-motif-related domains (Table S4).

**Figure 5:**
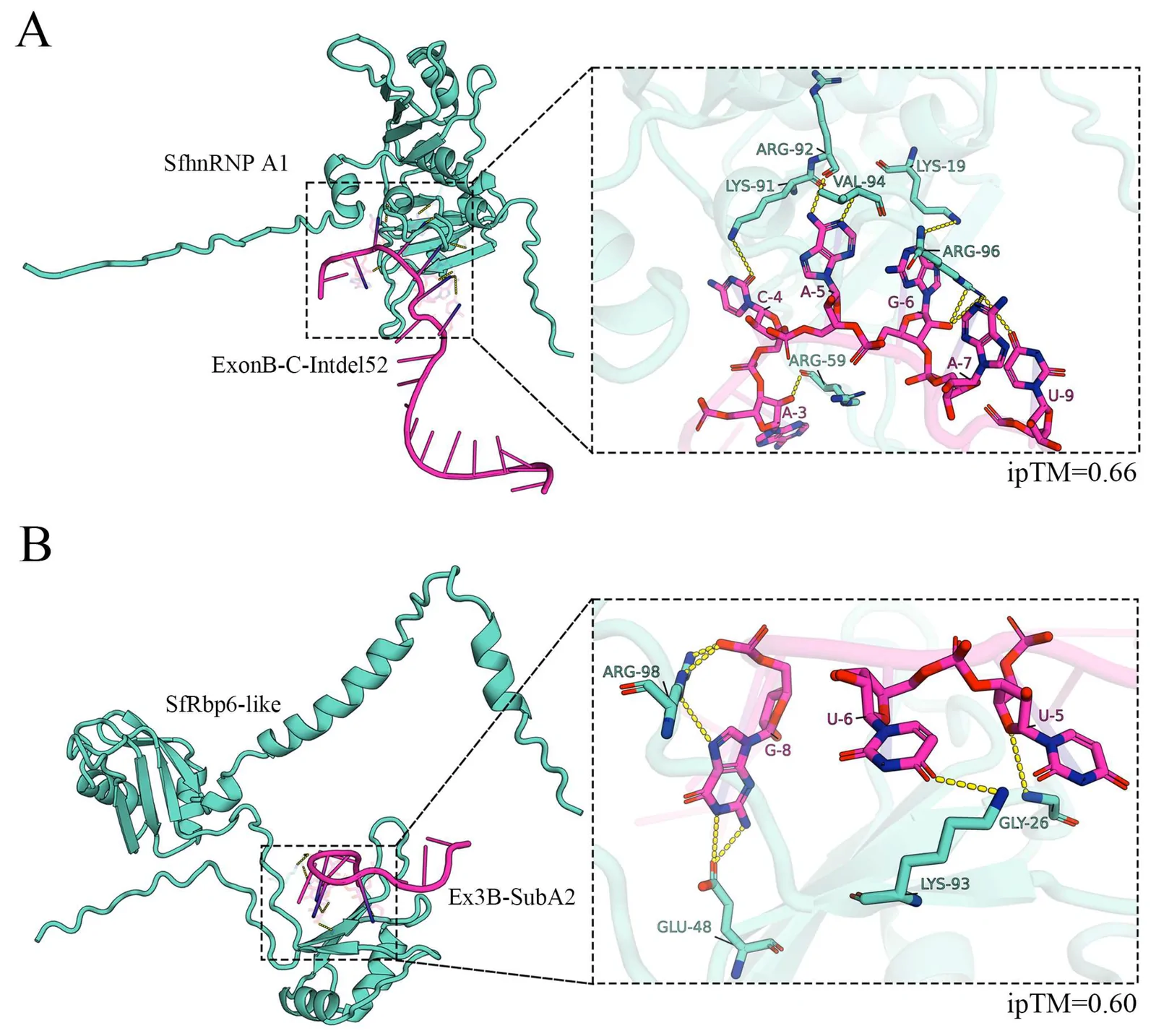
AlphaFold3-based protein–RNA complex prediction of SfRBP homologs and the corresponding *SfGluCl* candidate *cis*-regulatory RNA regions. The predicted complexes had ipTM values of 0.66 (**A**) and 0.60 (**B**). SfRBP homologs, *cis*-regulatory RNA regions, and hydrogen bonds are shown in cyan, magenta and yellow, respectively.

Based on motif prediction, BLASTP homology, and conserved domain annotation, two representative RBP homologs and their corresponding RNA fragments were selected for AlphaFold3-based complex prediction. The SfhnRNP A1-like (XP_035441205.1) was modeled with the RNA fragment corresponding to the region deleted in the ExonB-C-Intdel52 construct, and the SfRbp6-like (XP_035437037.1) was modeled with the Ex3B-SubA2 construct. The predicted SfhnRNP A1-like–RNA, and SfRbp6-like–RNA complex showed an ipTM value of 0.66 (Figure 5A) and 0.60 (Figure 5B), respectively. In the predicted interface, multiple hydrogen bonds formed with the RNA fragment, including Arg59–A3, Lys91–C4, Arg92–A5, Val94–A5/G6, Lys19–G6, and Arg96–G6 for SfhnRNP A1-like, and Gly26–U5, Lys93–U6, Glu48–G8, and Arg98–G8 for the SfRbp6-like. These AlphaFold3 models suggested that the SfRBP homologs could form structurally plausible interactions with the corresponding RNA fragments, providing hypotheses for future biochemical validation.

## 4. Discussion

In this study, the distinct stage-specific expression dynamics of the splice variants (*SfGluCl 3A*, *SfGluCl 3B*, and *SfGluCl 3C*) were detected from egg to adult. Their relative mRNA expression level generally declined from early to late instar larvae. Expression levels were comparatively elevated during the late larval, pupal, and adult stages. This temporal expression pattern is similar to that reported for *MdGluCl* and *Chilo suppressalis GluCl* (*CsGluCl*) [6,18]. The downregulation of three *GluCl* splice variants during larval growth may reflect a reduced demand for GluCls, whereas the increased expression in adults may correspond to physiological requirements, such as neuromuscular coordination or sensory perception as reported for *MdGluCl 3C* in housefly appendages [18]. Notably, the broadly similar inclusion patterns of the three splice variants across developmental stages suggest that *SfGluCl* exon 3 splicing may be maintained within a relatively stable range across the developmental stages of FAW. This stable inclusion profile is consistent with a developmentally persistent, constitutive-like mode of *SfGluCl* exon 3 regulation. Rather, it does not suggest pronounced stage-specific shifts in splice-site selection. Nevertheless, since this analysis was performed using whole-insect samples at different developmental stages, tissue-specific or condition-dependent regulation cannot be ruled out. The broadly stable inclusion pattern nevertheless motivated us to investigate the *cis*-regulatory elements underlying *SfGluCl* exon 3 MEAS.

From the perspective of pre-mRNA splicing mechanisms, recognition of eukaryotic introns requires several core splicing signals, including the 5′ splice donor site, branch point, polypyrimidine tract, and 3′ splice acceptor site, and is also subject to fine regulation by classical auxiliary cis-elements, such as exonic/intronic splicing enhancers and splicing silencers [26,27]. In this study, intron-deletion identified intronic regions affecting exon 3 splice variant selection, and subsequent intron-deletion analysis identified the 20 bp region deleted in ExonB-C-Intdel52 as a candidate *cis*-regulatory region. Because the minimal functional sequence and responsible nucleotides have not yet been defined, this region may encompass one or more *cis*-regulatory elements.

In *SfGluCl*, the 20 bp candidate intronic *cis*-regulatory region deleted in the ExonB-C-Intdel52 construct is located 12 bp downstream of the 5′ end of the intron. Deletion of this region increased *SfGluCl 3B* inclusion by 52.97% points and decreased *SfGluCl 3C* inclusion by 47.58% points. Similar intronic *cis*-regulatory elements located near 5′ splice sites have been reported in other alternative splicing events. Examples include ISS-N1 in the downstream intron of *SMN2* exon 7 [28] and an element adjacent to alternative *FGFR2* exon IIIb [29]. However, even among elements located near the two ends of an intron, differences in spatial distribution may suggest different functional hierarchies in the regulation of MEAS. Specifically, the candidate intronic *cis*-regulatory region in *SfGluCl* starts at 12 bp downstream of the 5′ end of the intron. Although it lies in the proximal region of the 5′ splice site, its separation from the splice donor site suggests that it is unlikely to constitute the 5′ splice donor site itself. Instead, it may influence exon 3 splice variant selection by providing a potential binding site for RBPs involved in pre-mRNA splicing, such as SR proteins or hnRNPs [10,11].

Substitution of six nucleotides within an 11 bp candidate exonic *cis*-regulatory region located within exon 3B, away from the splice-site boundaries (internal exonic *cis*-regulatory regions), increased *SfGluCl 3B* inclusion by 66.14% and decreased *SfGluCl 3C* inclusion by 63.52%. These results suggested that this exonic *cis*-regulatory region may contain a candidate *cis*-regulatory element involved in exon 3 splice variant selection. Because exonic *cis*-regulatory elements are often short and may overlap with coding sequences, this substitution could potentially disrupt exon 3B-derived ESE/ESS-like motifs and/or introduce exon 3A-derived ESE/ESS-like motifs. Similar phenomena are also reported in alternative splicing events of other genes. For example, a single-nucleotide mutation within *SMN2* exon 7 can markedly alter exon 7 inclusion efficiency [30]. Mutations in internal exonic *cis*-regulatory elements of *BRCA1* and *BRCA2* can lead to aberrant splicing [31]. Internal exonic regulatory elements in *CFTR* exon 9 can affect exon recognition and inclusion through splicing factors, such as SR proteins or hnRNPs [32]. These studies indicate that internal exonic *cis*-regulatory regions are important sites for the regulation of alternative splicing.

Because this internal exonic region is located within the exon 3B, the substitution also affects codon usage. Exonic nucleotide substitutions, including synonymous codon changes, may influence pre-mRNA splicing by disrupting exonic *cis*-regulatory elements or other local RNA-level regulatory features [31,33]. According to the predicted coding frame, four of these codon changes are synonymous, whereas one leads to a conservative amino-acid substitution from Ile to Leu. The effect of Ex3B-SubA2 was measured by RT-PCR at the transcript level. Therefore, the observed change in exon inclusion most likely reflects altered local RNA-level regulatory information, such as ESE/ESS-like motifs or other sequence-dependent splicing features. It is unlikely to be a direct consequence of the amino-acid substitution. Nevertheless, this coding change should be considered when interpreting the functional implications of this candidate exonic *cis*-regulatory region. One possible explanation is that the candidate exonic *cis*-regulatory region affects the exon-definition process. Unlike sequences located directly at splice-site boundaries, this internal region does not constitute the 5′ or 3′ splice site itself but may contain binding sites for exonic splicing enhancers or silencers. Recruitment of corresponding splicing factors could alter recognition of exon 3B and its competitive relationship with exon 3C. However, the presence and identity of such binding sites remain to be validated experimentally.

Conservation analysis across the 24 lepidopteran species showed different patterns for the two identified candidate *cis*-regulatory regions (Figure S8). The 20 bp candidate intronic *cis*-regulatory region deleted in the ExonB-C-Intdel52 construct was not broadly conserved, suggesting that this intronic *cis*-regulatory region may be lineage-specific or rapidly evolving. By contrast, the exon 3B region modified in the Ex3B-SubA2 construct, as well as the corresponding region in exon 3A, was highly conserved among the analyzed species. Because the exon 3B region modified in Ex3B-SubA2 is located within a coding exon, its conservation may reflect protein-coding requirements, splicing regulation, or both.

**Figure 6:**
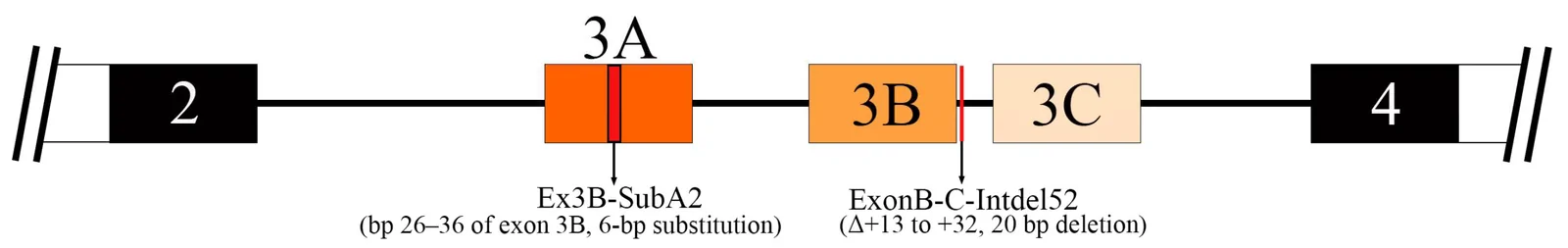
Schematic summary of the candidate intronic and exonic *cis*-regulatory regions corresponding to ExonB-C-Intdel52 and Ex3B-SubA2, respectively. Black boxes indicate the constitutive exons 2 and 4, and red, orange and light-orange boxes indicate the mutually exclusive exon 3A, exon 3B, and exon 3C regions. Red vertical lines delimit the regions modified in the Ex3B-SubA2 construct and deleted in the ExonB-C-Intdel52 construct.

RBPmap and ATtRACT predicted hnRNP-related motifs within the two-candidate *cis*-regulatory regions. AlphaFold3 modeling further suggested that corresponding FAW RBP homologs could form structurally plausible contacts with the relevant RNA fragments. These computational results support the hypothesis that these candidate *cis*-regulatory regions may act through RBP recruitment. However, neither direct RBP binding nor the functional involvement of the predicted RBPs was demonstrated in this study. A schematic summary of the candidate intronic and exonic *cis*-regulatory regions is shown in Figure 6.

Although insects typically possess a single *GluCl*, AS generates diverse splice variants, thereby expanding the functional diversity of GluCl subunits. Among these, AS events involving exons 3 and 9 are particularly notable because of their potential functional significance [19]. Exon 3 of *GluCl* belongs to a cluster of mutually exclusive alternative exons and encodes part of the N-terminal ligand-binding domain near the conserved Loop D region, suggesting that AS at this site may influence ligand recognition or receptor pharmacological properties [34]. For instance, three exon 3 splice variants of *MdGluCl* from *M. domestica* showed similar sensitivity to glutamate and ivermectin, yet the *MdGluCl 3A* and *MdGluCl 3B* displayed higher sensitivity to fipronil and picrotoxinin compared to *MdGluCl 3C* [18]. Similarly, the *AgGluClb1* from *A. gambiae* showed reduced sensitivity to picrotoxin and lindane [20]. In *C*. *suppressalis*, the inclusion of exons 3A and 9C negatively correlated with resistance to emamectin benzoate, whereas the exon 3B- and exon 9A-containing splice variants were positively correlated with resistance [35]. Collectively, alternative splicing of *GluCl* exon 3 influences the pharmacological properties of insect GluCls, providing a functional rationale for investigating the regulatory mechanisms controlling *SfGluCl* exon 3 splice variant selection. Therefore, the candidate *cis*-regulatory regions identified here provide molecular entry points for future functional studies, but they should not be interpreted as direct evidence that *SfGluCl* exon 3 splicing alters insecticide sensitivity or receptor activity.

However, limitations should be noted. RBP motif and AlphaFold3 modeling were only hypotheses and the responsible nucleotides, direct RBP binding, and underlying mechanisms remain to be validated. Future studies should use isoform-resolved quantification, targeted mutagenesis, RBP-interaction assays, and in vivo functional validation.

## 5. Conclusions

In summary, this study identified one candidate intronic *cis*-regulatory region deleted in the ExonB-C-Intdel52 construct and one candidate exonic *cis*-regulatory region modified in the Ex3B-SubA2 construct that should contain a candidate *cis*-regulatory element which can influence *SfGluCl 3B* inclusion in an Sf9 cell-based minigene system. These findings provide initial evidence that both intronic and exonic sequences are associated with splice variant selection of *SfGluCl* exon 3. Importantly, the candidate *cis*-regulatory regions identified here provide molecular entry points for future functional genomics studies to determine whether modulation of *GluCl* exon 3 inclusion affects receptor properties, insecticide responses, or neuronal physiology, and whether RNA-based manipulation of splice variant selection has potential as a future pest-management strategy.

## Data Availability

The raw data supporting the conclusions of this article will be made available by the authors on request, as the data are part of an ongoing study.

## Supplementary Materials

The following supporting information can be downloaded at: https://www.mdpi.com/article/10.3390/genes17080899/s1, Table S1. Lepidopteran species used for comparative sequence analysis. Table S2. Primers used in the present study. Table S3. Stepwise intronic deletion strategy and selection rationale for *SfGluCl* exon 3 splicing analysis. Table S4. Candidate RBP-binding motifs in ExonB-C-Intdel52 and Ex3B-SubA2, corresponding *S. frugiperda* homologs, conserved domains, and AlphaFold3 protein-RNA complex prediction scores. Figure S1. Cycle-number optimization for semi-quantitative RT-PCR. Figure S2. Representative restriction-enzyme digestion patterns used to distinguish *SfGluCl* exon 3 splice variants. Figure S3. Intron-deletion constructs of Intdel1. Figure S4. Intron-deletion constructs of Intdel2. Figure S5. Conserved sequence cluster in the intron between exon 3B and exon 3C. Figure S6. Intron-deletion constructs of Intdel3. Figure S7. Intron-deletion constructs of Intdel4. Figure S8. Conservation analysis of the ExonB-C-Intdel52- and Ex3B-SubA2-related regions across 24 lepidopteran species. Text S1. Amino acid sequences used for AlphaFold3-based protein–RNA complex prediction. Text S2. The *SfGluCl* exon 2–4 sequence derived from the genome assembly. Text S3. The *SfGluCl* exon 2–4 sequence obtained by experimental sequencing. Text S4. pMIB/V5-HisB.
