# Supplementary material for: Identification of Cis-Regulatory Elements Involved in Mutually Exclusive Alternative Splicing of Exon 3 in SfGluCl from Spodoptera frugiperda
Source: Genes (Basel). 2026 Jul 30;17(8):899. doi: 10.3390/genes17080899 (PMC13512814; doi:10.3390/genes17080899)
Supplement: Supplementary file 1 [file genes-17-00899-s001.zip › Supplementary Information-Sf.pdf]

Supplementary Information

The supplementary information includes the following contents:

Table S1. Lepidopteran species used for comparative sequence analysis.

Table S2. Primers used in the present study.

Table S3. Stepwise intron-deletion strategy and selection rationale for *SfGluCl* exon 3 splicing analysis..

Table S4. Candidate RBP-binding motifs in ExonB-C-Intdel52 and Ex3B-SubA2, corresponding *S. frugiperda* homologs, conserved domains, and AlphaFold3 protein-RNA complex prediction scores.

Figure S1. Cycle-number optimization for semi-quantitative RT-PCR.

Figure S2. Representative restriction-enzyme digestion patterns used to distinguish *SfGluCl* exon 3 splice variants.

Figure S3. Intron-deletion constructs of Intdel1.

Figure S4. Intron-deletion constructs of Intdel2.

Figure S5. Conserved sequence cluster in the intron between exon 3B and exon 3C.

Figure S6. Intron-deletion constructs of Intdel3.

Figure S7. Intron-deletion constructs of Intdel4.

Figure S8. Conservation analysis of the ExonB-C-Intdel52- and Ex3B-SubA2-related regions across 24 lepidopteran species.

Text S1. Amino acid sequences used for AlphaFold3-based protein-RNA complex prediction.

Text S2. The *SfGluCl* exon 2-4 sequence derived from the genome assembly.

Text S3. The *SfGluCl* exon 2-4 sequence obtained by experimental sequencing.

Text S4. pMIB/V5-HisB.

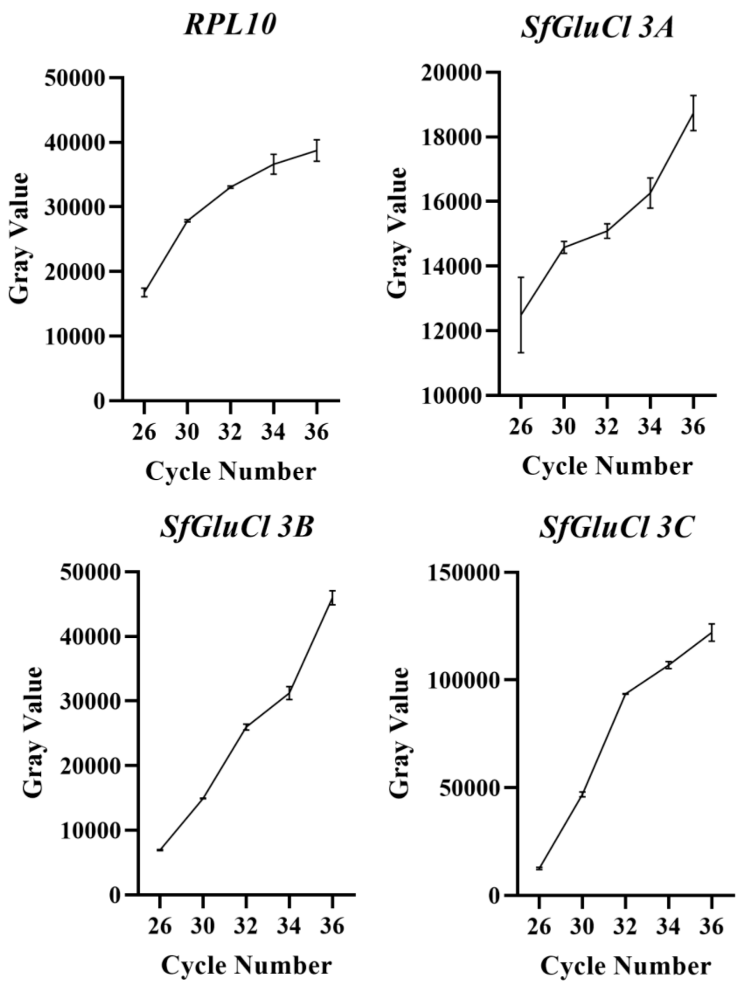

Figure S1. Cycle-number optimization for semi-quantitative RT-PCR.

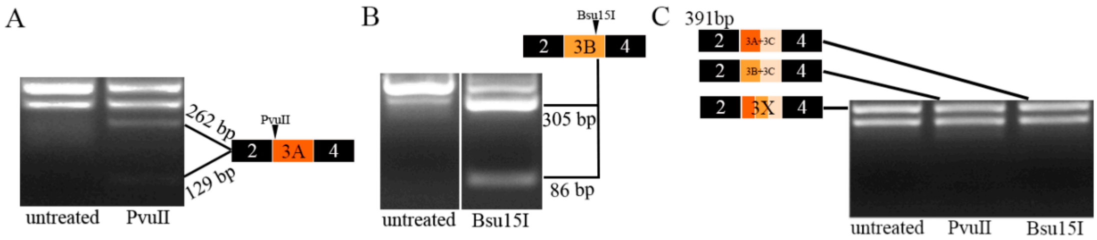

Figure S2. Representative restriction-enzyme digestion patterns used to distinguish *SfGluCl* exon 3 splice variants. (A) PvuII digestion of *SfGluCl 3A* generated 262- and 129-bp fragments. (B) Bsu15I digestion of *SfGluCl 3B* generated 305- and 86-bp fragments. (C) *SfGluCl 3C* lacks both restriction sites and remained within the intact band after digestion; its inclusion was calculated by subtracting the quantified *SfGluCl 3A* and *SfGluCl 3B* products from the total undigested product. Variant identities were confirmed by Sanger sequencing.

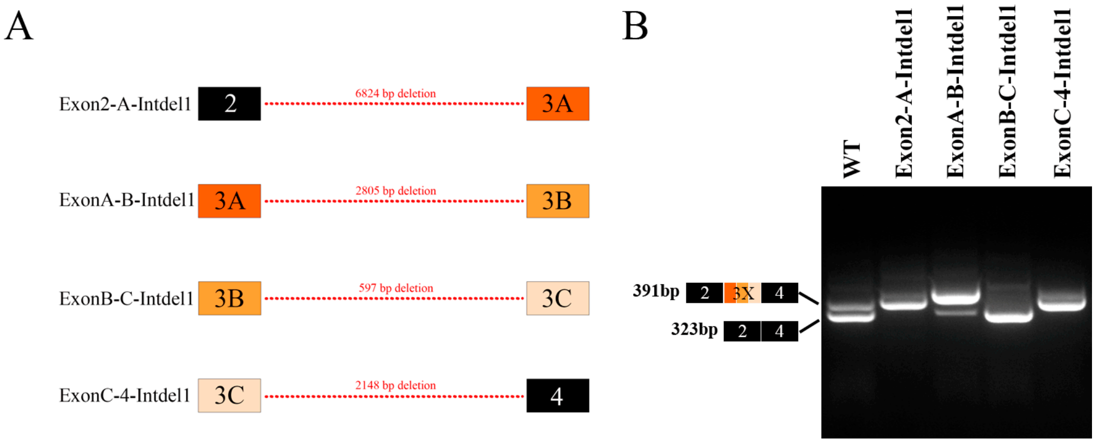

Figure S3. Intron-deletion constructs of Intdel1. (A) Diagram of four intron-deletion constructs. (B) RT-PCR assays of four intron-deletion constructs.

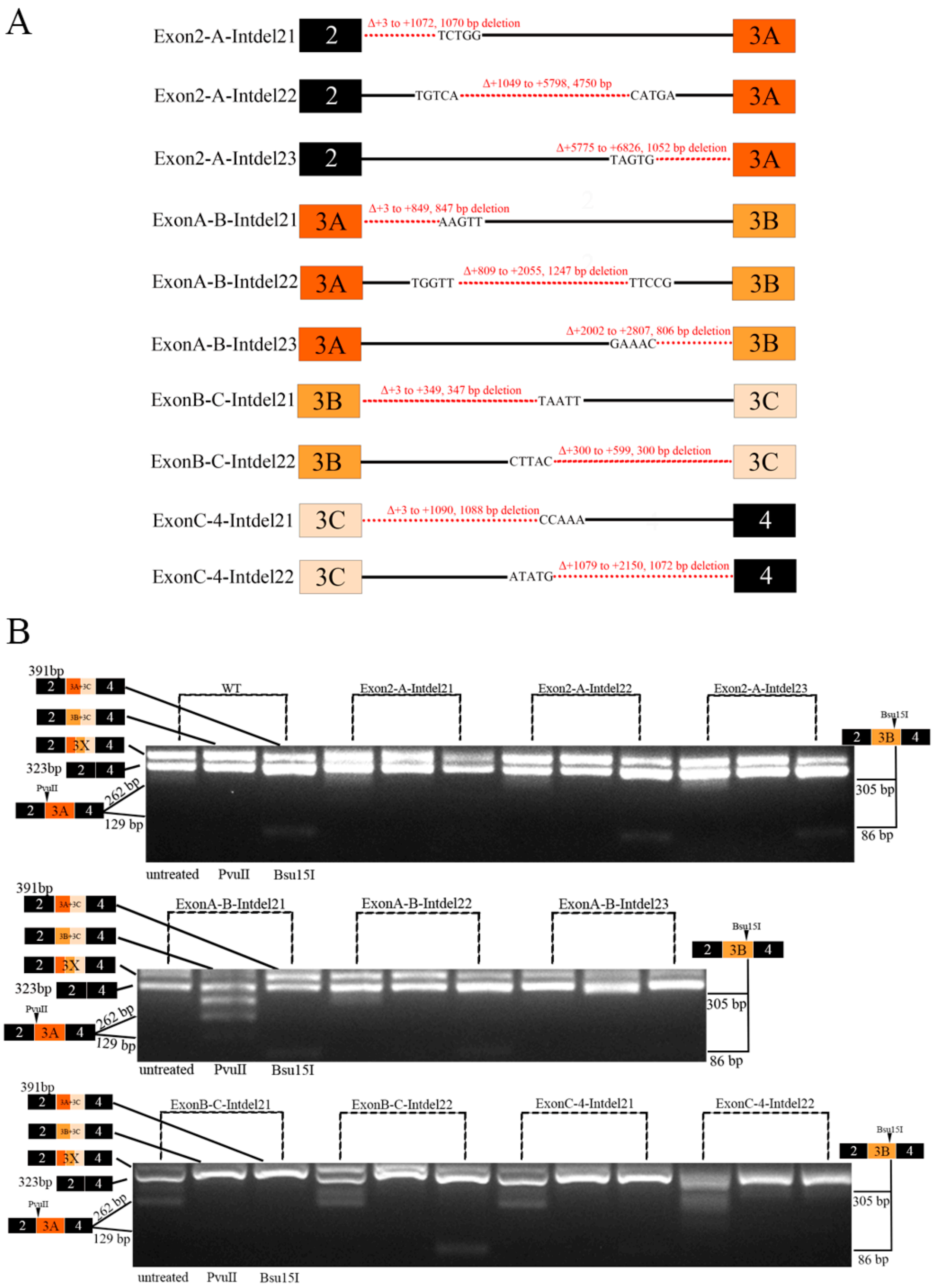

Figure S4. Intron-deletion constructs of Intdel2. (A) Diagram of ten intron-deletion constructs. (B) RT-PCR assays of ten intron-deletion constructs. Deletion coordinates are numbered relative to the first intronic nucleotide downstream of the upstream exon.

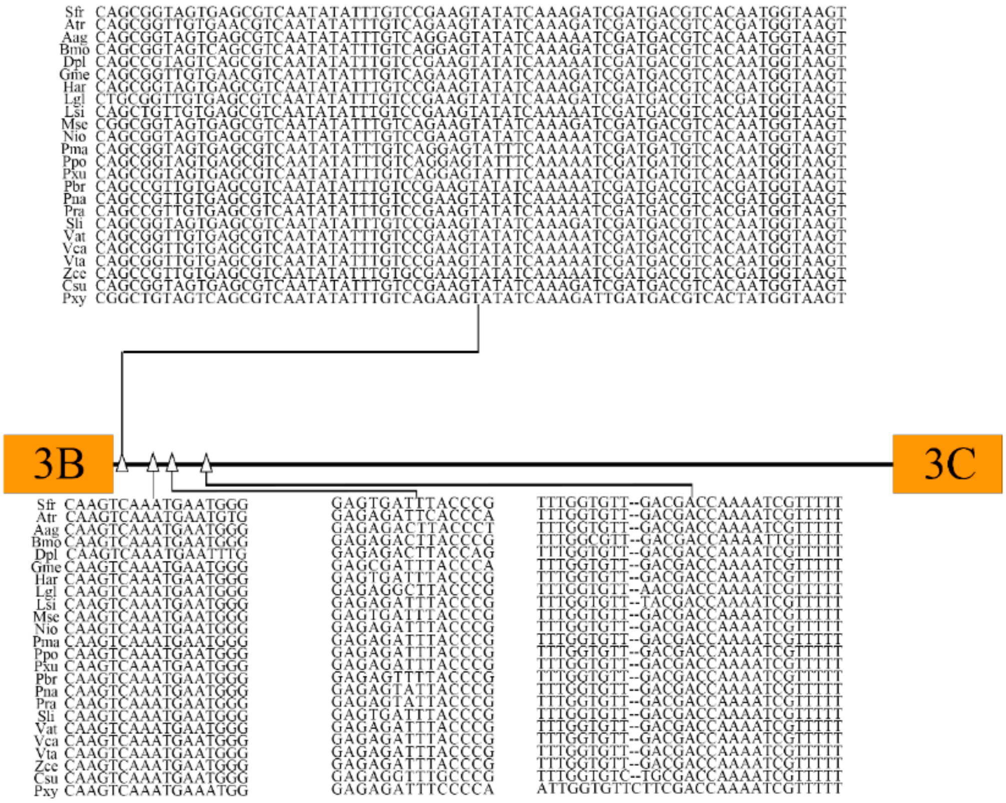

Figure S5. Conserved sequence cluster in the intron between exon 3B and exon 3C

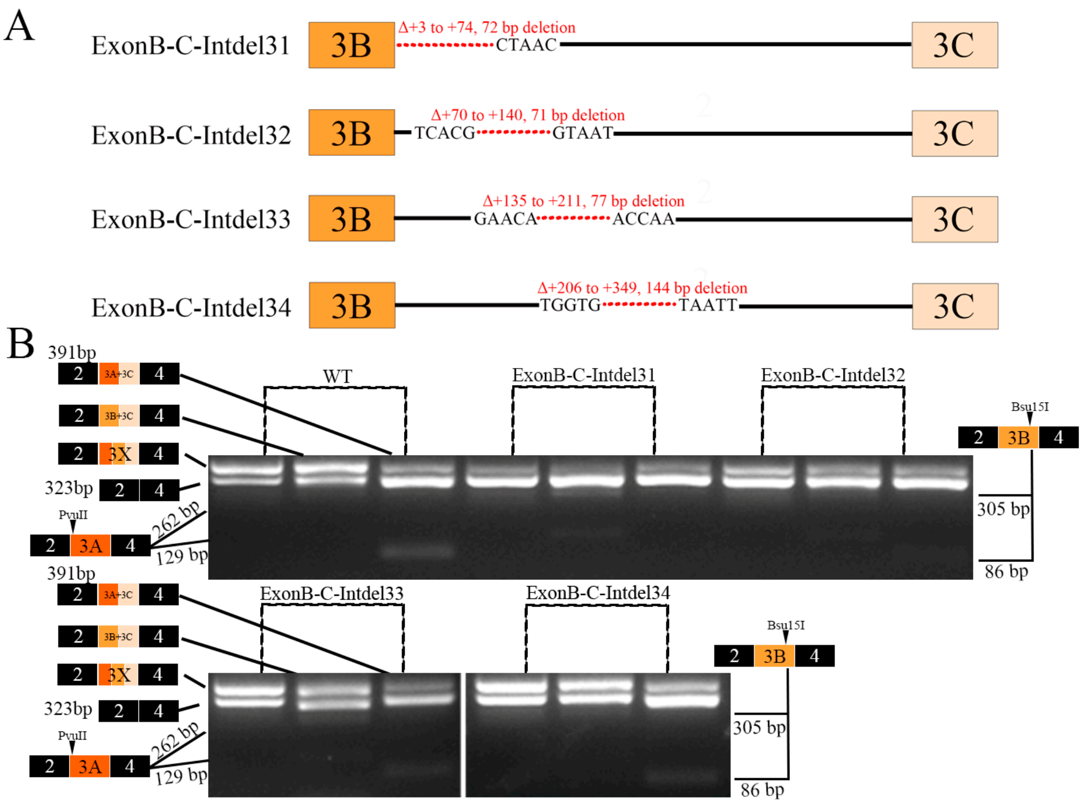

Figure S6. Intron-deletion constructs of Intdel3. (A) Diagram of four intron-deletion constructs. (B) RT-PCR assays of four intron-deletion constructs. Deletion coordinates are numbered relative to the first intronic nucleotide downstream of the upstream exon.

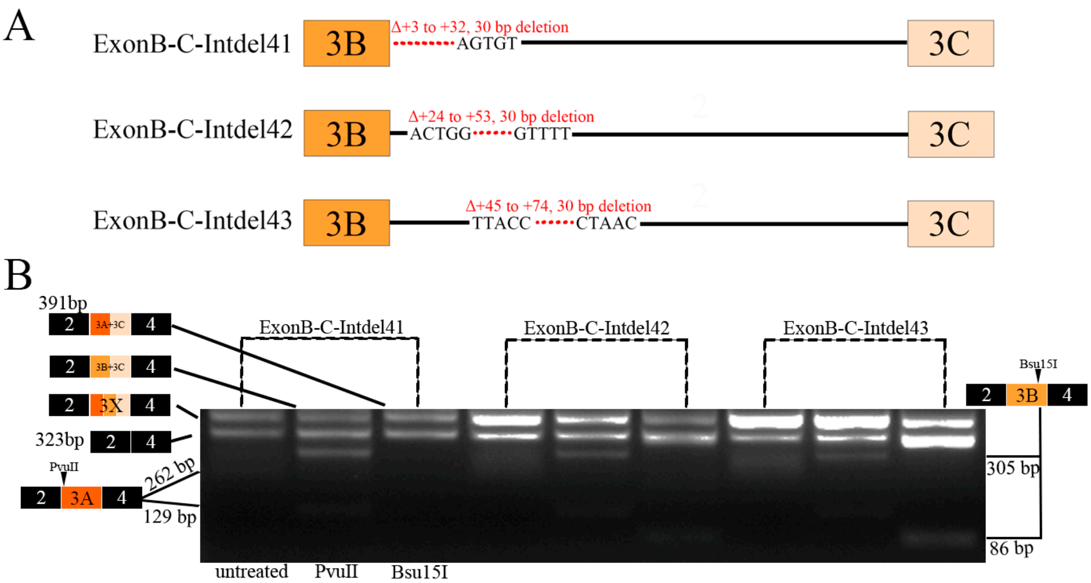

Figure S7. Intron-deletion constructs of Intdel4. (A) Diagram of three intron-deletion constructs. (B) RT-PCR assays of three intron-deletion constructs. Deletion coordinates are numbered relative to the first intronic nucleotide downstream of the upstream exon.

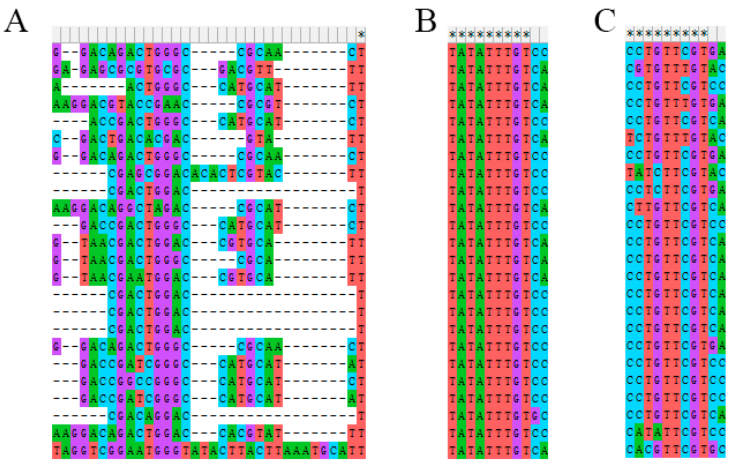

Figure S8. Conservation analysis of the ExonB-C-Intdel52 and Ex3B-SubA2-related regions across 24 lepidopteran species. (A) Multiple sequence alignment of the 20-bp intronic region corresponding to ExonB-C-Intdel52. (B) Multiple sequence alignment of the Ex3B-SubA2 region within exon 3B. (C) Multiple sequence alignment of the corresponding region within exon 3A.

Text S1 Amino acid sequences used for AlphaFold3-based protein–RNA complex prediction

>XP_035441205.1 SfhnRNP A1-like

MKPSGGGGDDYEEPEHLRKLFIGGLDFRTNDASLKEFYEQWGEIVDVVVMKDPQTKRSRGFGFITYSRA
HMVDDAQKNRPHKIDGRVVEPKRAVPREEIKRPDAAATVKKLFVGGLKQDIEEEDLKEYFSSYGNIVSVS
LVTEKDTGKKRGFGFVEFDDYDPVDRICLHPNHKVKGHRLDVKKALSKSEMAAGGPGGGGSSGGGGGR
RGGGRGGG

>XP_035437037.1 SfRbp6-like

MDTQNQDVARSPAEVPNDPGKMFVGGLSWQTSPESLRDYFSKFGEITEVMVMKDPTTRRSRGFGFITFGD
PASVDKVLAQATHELDGKKIDPKVAFPRRAHPKMVTRTKKIFVGGLSAPTTLEDVKNYFEQFGPIEDAML
MFDKQTNRHRGFGFVTFQSEDIVDKVCEIHFHEINNKMVECKKAQPKEVMLPANLAKTRAAGRGAYDF
MWSLGALPDGFPAAYAAYAAGRSYSGY

Text S2 The *SfGluCl* exon 2-4 sequence derived from the genome assembly

Note: Exon sequences are shown in gray; the rest represent intronic sequences.

ATGTATGAACGGTGGGAAGATAAATTTCCGAGAGAAAGAGAAGCAGATCCTCGATCAGATCCTGGGTC
CTGGGCGTTACGACGCCAGGATCCGACCTTCAGGCATCAATGGCACTGGTAAGTCCTCGCTTCGCTTA
CAGCTTCATTCGGTCCGTGGTGGAAGCGATAGCTTAGGATCTAGGGAGATAGGATGGTCTAGGGAGAT
AGGAAGAGTAGATGGACAGGTCTGAGCAAAACTGTGGTCTCAATTTTAGCAGGTCGTAGAGGATAGG
TAGTGGATCTGGTCAGAACTTTAACATAAATGGAAAAAGACCAGGAGAAAAAAGTAGAATGGATGAC
TCAACAGATATTTTTAATACAATTTTATAGTTATTTGTTACCAACGAGGTCGAGTGGTTGCAAGTGCGAC
TGCTGGACAAGGGGTCTCGGGTTTGATTCGGGTTGGGCAAAGCATTACTGGGCTTTTTTCGGTTTTTCG
AAAATTTCTCAGTAATAGCACAGAGTCTGGAATTGTGTCCAGTATATGGCAATGGGCTCACCCCCTATT
ACATAACACAAATTGTGAAAAGTGGGTGTACATTGTATAGCGGCATTACGTGCCGTAATGCACACCTCT
GCCTACCCCTTCGGGGATAAAAGGCGTGACGTTATAGGAGTTTTATAGTTACAAATCTTTGTTTAGGAA
TCATGAAATAACAAAACATTACAGCTAAATGTCTGTAATGTGAAATCATAATCGTAAAACGTGAGAACA
CAATAAACTTGATTAATGTTGTAAATCATGAAGTAATAATAGACACAGACACATTTATTTTGACACACAT
CTGTGCAAAACATGTGTTGTCTTGGCTATCAGCTATTTATGGAACTAAAATATATTTTAGTTGCCACCAT
TATTAATAGAGTTACTGCTCTTATATTTGTCTCGTTCGAAAATAACGGTCGAATTTATTATTGGCGGCCAT
TTTTTATTTTGGAGTCTTTGGCGAAGATTTGTTGTCGTAGGACAATTTAGTAAAAAGTAGGTACTTTAGT
GATAAAAGAAGTTTTTATTGGTGAAAGTTTTTTTTTGGAAATCGGTTTTTAAGACGAAATTTTGCCAGT
TTAATGTGTTTTATTTTCAATGACAACTGCACGGTTGGCGCGGTGGCTGGGCAACTGGCTGCCGCACA
ATGGGTAGCGGGTTCGATTCCTGAACGGAGCAACTCTTTGTGTGATCTATAAATTGTTGTTTCGGGTCA
CATGTGTATGTGAACTTGTATGTTTGTGAACGCACCCCATGACACTGGTGAAAATCCTAGTGTAAAAAT
TAAAAGGATTTGTCTTTTATAACTTAACAATACACCTGACCAAAAATAAACGAGATCCTCTTATACAATC
TCTTGAAAAACTAAGGTCAACTAAAAATAAGTTAAAAAGAAAACCACTGACCTTACAATTAAATACTA
AAAACGTATTTTTATATAAAGGAATGATGATAAATGTATTTTTTATTTAGAAGCACTTAGTGCTAAGATCT
GTACCTTTGTGAGGAAGATATAGAACGTTGAGAATTCTAAGGGTCTAACAAAAGACGGCCGCCATCTT
GGAATTGCCACGACAATCATTTATCGTCGGTTTTCATGCCAACTGTTGGCACCTTCTGACAATTTTCTCC
GAGATAAATAATAACACTGTTATGTCTCTAAGTTTGCCTTTTGAAATGTTTCCCTTTGTCTCCATGTACT
GATTCTAGATGTTTTAATGTTTGTTTGTAAGCTTTCTTTGACGGGAAAAGTGTTTGCTATACATTCAGTT
TTTCTAAATTACTGCCTCACACAGCAAAATTGTGGAATGTTCTGACGTCCGTGGTATTTCCGAACCGAT
ACAACCTTCATACTTTCAAGAAGAAAGAAAATAGCGTATTCCTTTTTAAAAGGCCGGCAACGCACCTG
TGACTTCTCATATGCTAGTTTGCTTCCCATTGCATAAAAAAACTTTTACTTATCTCTGCGCGAAATAATTC
ACATTATAATACTATAATCACATTAAGAAAGATCTCTAAACATATAACACAATAGATTGAATAGCAACCA
AATCCTATATTTACCGACACACAAAACGTAATCCCTTCAGACCCACCACATCGTCTGCCAAGTCAAATT
CGTAGTTGCATGACAAACGCGTCTTCCTAATGGTGCCGTAATGTTTGTGCAAACAACGTTGTCTGTCCA
TTAGTCACTATTTACTTCACCTGCTCTTCTCCCTTACATATATAACATACTAGCTTCTGTCAACGGCTTCA
CTCGCATTCCCATGACATAAAATATAGCCTATGTGTTATTCCAGACCGTAATATGCCCCTGTTTTTCGGA
GCTGCGGATTACCTAGCGGGTTTACCGGGGCTCCGGCTCGAAAAGCAGGAGTAGGAACAGGGTGGTT
TTTAATCATCAAGAGTCTGACACTCTTACTCGCCTCACCCAAGGAAGAAGTCATTGAATGATTTTTCAA
AAACCCCTGATTCAACCATGTAGACGTGATTGAGTAAAAACATATACACAAACTTACTCATAAACTTTA
TTAGTAAGATGGCAAAGTTTTCTACATATTATATGCTTTGTATATCACCCCTACATTTGGCACTAACATCG
GCGGATGTGTTTAATAGATTCTTGGCATTTGTACATCTGATTCAGGTGCTCTCTCATCATAGTTTATTAGC
CCTTTCAAGTCAGACAGTAATTTGGTAGTTTGTTATTGCAATATGATTGGGTATCTGGCTACAGATGTTT
CTCTGTCTACGCTCTTAATAATATAAGATTTTGAAAATGTCTGTTGAAGTAATCACTGTTAATGAAATGC

TATAGAATATTCATTTGATAACTAAAGTTTCTTCTTTGATTTAGTGGTATAGGAGAAAAGAACTGTAATAT
ATTATCTAGAATCTAGAATATCTTTAGGTATCTGAAAAAGCTATCGCTTCCACGACATTAAGACCACAGC
TAATTCTAGTTACTTAGAAAACACTAACTGTCAACGTTATGATATTAACAAACCGATAAACTTGCTGATT
GAATTATAATTTTGTTTTCAAACTGAAAAGATAGAAACAGTCGCTCACGATGTATATAAATATCAACTTT
TTTCTTTTATTTTATGTAAATGACTATGCTATTTTTAATTTTGTTCGAATAGTTTTCTCAAAAAACTTTAAG
GTCGCAAAAAATATACTTTCTTAACTAACATACATATAAATAAAATTCGCAAACCAACTGGCTCTTTAGT
TACAGAGATAATAAAAAAGAAATAAAAAGTCACCGCGGTGCGTTTTGTGAAAGATTTAAAAGCAACG
CTTTGCCAAATTCTGCATCGATAACATTTACTTACAAAATGTATTGCAATGAATATATTAGAGACTGGAA
TGTGCATACCACATGTACACACAAGTACCTACCCACGGCGGGAAGAGAACGCGACTTAAAATAAACTA
CGGATTGTGCGCAAACTGCTAGTGTAGGCTGAATGTCGTAATCCGAGTAATTTGTTACGTTTTTGTTGT
CTTCTGTTAATAAAAGTTCAATTACTGCAGCCAATGTTTCCTCCGGTCAATACTTTTGGCGTCACTCCAA
ATATTTTCTAGTTACATATTTAAAACTGTCAAACGATTTATTTGGTATGCGATCTTTGCCCTTTCTGACTT
TCTTATTGATTTGTTACAAATATATAGTTATTATATTATTATTCTCTAACAAGATATGTTATATTCATGAATT
CTTTTCATCTTCGTCAATGTGAAATAGTGTATCTGTTTATTCATTTATTCTGTAGGTCACATCTAGTAGGT
CGTGCTCACCTCTTTCAATATCCATGTCAAAAATCCGTCTTTTTCGTATTGTGTATTGAAGCGTAGTCAT
TTTTCTATAGTGTGTGTGTTGTTTGAAAGGTTTTTATCTAATATCTTGGCTATTAAAGGGAAGTCGTTTGT
CTGGCTGTCGGAACACATTAGCTTGATTCTTGATATTGTGGGGAAAAACATCGTAACGTTATTTGTACT
CGTACTACCTATGTATGTGTATTTTATTCCTCATAGGTGCGATCGATTTCTTTACGAAGACGTTTCAGATT
ATGTTATTATTTCAGAATGAAGATATGCATAAAATATATTTTGTTTACTAATAATGTTTCACAGGAAACTA
GGCATAATAAAACCACAGGCACCACTATTATTATTTCCTGTGGCCATAGATTACATCACGCTGTTCATTAT
CTAGAATATATTAAGATATCTCAACGAGATTGGTTTTCTCGAGAGATACACAAAAAAGCATCGGAGTGC
CACTCAAGTGCTACATCTCGTAATTTCCACAAGTTCATTGCGTAACAAAAGAGGAGCGTTACGTGAAA
ACCATAAATCACGCCGCGATTTTTTTCCGCCATCTTTATTTTTACAACTTTGCGCGGGAACGCGGGAAT
CTGTAATGGAAGAACATTTCACGGAGCCTCCCTGAGCGTGATTTTTATGATTCTGTACAGTTTTCTCGTT
AATGGAAATGGCATTTACACTTTAAGAGGTTCGTGGAAATGTGCAAGTCAGAGGAGAGGCTTGTAACC
TCTTTGCTTCCCTTTATTTAGATATCTATTGGCTAACAGTCAGTTGCAGAAAACTGTCTTTAAATTCGAC
ATTAGTTAATCCGTCATTATCGCTAATATTCCATTAAATTTAAAGACGTTGCACAAAACTGTTGCGGGTC
CCTTTAATGGGACTTTAACTAAAGACGACATTAAAAAGTGTTGCAAGTTAAAACACATATTTTGAATGA
ACTGATTACGTAGTATTTGTATACTCTCTGGAACTGATTGAATGTAAATTGTCATTAAGGATGGAAGGAA
GCGAATCCCGCTTCGAAAGAAACCGATTTTTTAAAATGACGATTTATCGATTGTGTTTGGATTTATTTCA
TGTAGTTAAAGGACACTATAAAAATCAATACAATACCATTTGTTACTACTTAGTTACTTGTAAATTAAGA
CTCAGTTATGAGTTTTTTCAGAGCTAATTACATTATTTACGTAATAGCTATATCTATATTTTGTATTTGATTA
AATATCTCTATATTTGACAGATTAAGCATATGTGTTAATTTAATTAGCTACTATTAAATGTTTTTTGTAGTA
TCTTAATAGTGGAATACATATAAATATCAAATCTTATTCTAATTCACTGACCGATTCAATTATGTGTTCCG
ATTTAAAATAGGTATCAACCTTTTTGGTTCGCGGGAAGAAATTCCTAGATCGCCAGCCCTAGTTGTCAA
TATTGACATCATGAGCGTTTCTGTCATCTCTGTATTTAGGAAAAATCCAATTTTTTACGATAAAAATATTA
TCAATTGCTTTAATGTGGTGTTGAATTATCTTTCATTGGTATCGTAACAGCGTATTTAATATTTAATATTGG
TATCGACCAATATTCGAATTAATTATTTTCCGCCATTTTTAGGGTTCCGTAGCCAATGGCAAAAAACGGA
ACCCTTATAGATTCGTCATGTTTGTCTGTCTGTCTGTCCGTCCGTATGTCACAGCCATTTATTTCCGAAA
CTGTTGGGCCTACATAGTTGAAACTCTGTAGGTTGATGTATTTCGATAACCGCTATTCGAATTTTGAATA
AAAATTATTAAATTTAAAAATTAAAGGGTTTTTACATCCATACATGGAACTGTTTAAAAAAAATTTGTTT
CAGCTAACATACCCGTGATGGGTGTCTATGGATATGTCTTTTTTAAAGTAATGTCTTTAAAAAAGACATA
TCCATAGACACCCATCCAAAAGGTTCCCAAAACCATTTTTCGTCAAAATCAACCGTTTGAAAGTTAGA
CGCTTCCAAAGAGGAAAAATGAGTGCCCCCCCCCCCCTCTAACTTTTAAAATAAGAGAGTGAAAAAA

CAAAAAAAAATATATGGTGTAGATTTCAATAGAAACTAACAACGAAAATTGGTTTGAACGAGATATCA
AAAACCGTAAATATAATTTTGTCGTTCATACAAACACTATGTAAAACCAAGTTATTTTGTAACTTTTATA
ATATGTCATCTACTTGCTGTTACGGAACCCTTCATGGGCGAGTCCGACTCGCACTTGGCCGGTTTTTAA
ATATATCATTTGACGTTCCATGTCAGCTAACATTTATTTTAAACAGTTTATGTAGTCTATGAAATGACGAA
AAAGGTTTAATATGAAATGACGAAAAAGGTTTAATATATTTATTCCTATGAATAGTACTCAGTAATTAAAT
ACTATTATATTATAATTACATCCTACATTATATCTGTCTGGTGTTGCTAGTGGTTGTTAACACTAAGGAAG
GCCTATGTTTGGCAGGAAGCTGATCTTCGGCAGATCCAGTAGCGAAGAATCTCAAACATATCAATAACT
ATGTGAGGCCATCGATGGCTCTATTTCTTCGAGTTTGTTTGTAAATTGAGTATCTCGCAAATGTGTAAAG
CTTTAGAAATCCTGTATCTCACACGAAACTCTTCTCCGTCATGTTTTTCAAATAAATTTATCCTATCTGG
AATTACACGACGTCGTCGTGGAAGGAGCGGATAAAGATGAAAGGGACAAAATAATACTGTAATAAAGT
AAAAAAATATTACTAGCACTATTTACAAAAGAATTCAGAGTTTAATAACAAGTTTAATGGATGTTTGAC
TAGGCATTAAAAATTATAACCCCAAACTGTCAATTTAATGCTCCTTTAGCGCTAATGACGTTTTGTGCAA
CGTAAAAAATAGGCTAAAGTCCCGATTTGACGTTTCTTTAATTAAAGTTAATCCGGCATTAAAATGAGA
TAGAAATACTTCTTTGTGCAACACTTTAAAGCTTCATTAATATTTAAAGTCACTTTAAAAATTTAATGAT
CGTTCCTGCAACTGACTGTAAGAGATGAAGGCGAGTCTTTAGGGATACAAATAATTCAACTCAACGTG
TTATCTGACTGGTTCCGTCGCATTTGTCATCATCACGCTACTGACATTACAACCTTCCTAGATACTGTTC
CTTCAATGGAATCATATCCACAAATCATGGAACTCAATTTATCCATTACACTTGGACTTCAACCGTTTTA
TATACAAATGTGAGAGAAGTTATTCATCACTTAAGCTCTGTATGCGAGATTGGATAAGTGAGGTGGAGC
GCAAAATGACACCATCCTCCTTGATGAGATAAGAATAAGAATTGTAAAAAAATTGCAGGAAATTAATA
GCAATTCTCCCTGTTGTATTGTTAGTAATCGTTTGAGTGCTACGTTACATATAAAAGTCATAAACTCACG
TTGTAAAAGCGAATTTTATGAGATGTACTTAGCTCGGTTATACTCTTTAATATTGATGTTTGGAGTTGTAA
AAAGTAAGATAGTTCTATGTAAAAATAAACAAAGATCTATCCATAGAAACAGGATTTGTTGTTCCCTCT
TGATAACTTATTACTGACTAGTTTCAATAATTAAAAAACAAAGAGTAGAAGGACTATTCGTATATACGCA
ACGTAACGCCACACATTTTTATCCTCGACGGGGTAGGCAGAGGTGCACATTACGGCACGTAATGCCAC
TGTACAATGCACACCTACTTTTCACACATTTATATTATAAATCCCATGTAATAGACTTGAGCCTATTGCCA
TATACTGGGCATAATTCCATACTGCTATAACTAAATACCAATCTCATCAACATGCACTGCCAAATTGTTAC
CAGGTACATTTCTACGCTAATTATTTCCTCAACAATGTCCGCGATAAAATTAATCACATACGTTCCAAGT
TTCTTTTCATTCATTGTCGCCGACGTCTGCCACCATTGAATTGACCCCAAAAGGTTCCACAAAGGTTCC
ATTAATCACCCTTCTAATGAGTCAGGGTTGCCGCTTTTCTTAATCAATTATTCATGCTCTAAAAATATCAG
GTCCAGCTCGCTTCAGAGTCTTTGAAATACATAGATATTAAAGAGACTGTTGTTTTCTGTTTTCGTACA
ATTCGCGAGTGTTGATGAAAAGGTTATTTATTTTCTTTTCATGTTTTTTGAGGTTATTTTTTGCTAAATGT
GTTTTTGTAAGGTTGTGTAAGGAAGTGTGCTTTTGAAAGTTGTTTAGTGGGAAATTATGTAGGAATGTC
GTGTCATGAACAAAAAACAGCGCCTTGTGATCAGTCTTTGAAGTAATAACCACAAAGGTTAAGGATTT
TTCTCATTTGTAACGGTTTTTCTAGTAAGTTCTTTGTCTAAAGGTAAAATGTATTTTTAGGGAAGCAATA
ACAGAGTGTAGTTAGTCGCCAGCCCTGTTTATAAACATTCTATTTGCGGTGCTCGGCTGTCAAGTACAC
TGTGTACCGTATTGAACCATTTACAAGCAACTCACTTCTTTCTCCTTTGAAATGGCTTTATAAATAGTTT
ACCTTTTATTACACTTATATTTCAAATAGTTTATTGTTCATTAAACCTCTATTCTATATTGAAACTCAATAT
CAATCATTAATAATTGAACAAAGCATTATATTCTTTTTGTTTTCTCTCAAACTCTAGCCTATAATTAGGCT
GGAAATTTCTAGTAGATGTTTTCGATAAATTCATTGATTTCTAACCTATTATTAAACCTTATAGGTTTTTTA
CCATTATTATCCTTTAGAAGCGAGTAACAATATTAATAATAAACTATTAATTTCCCTTTGGCTACCTATTTA
CGTGAATAAACGTTGAATTATTGATGTTAACTTACACGCGAACGCGAACGTTAACCGTTTCCCAACACA
TTAGATTAAACTCAAATCGGTTTAACCTAGACATGTTTGGGAGCTATCGTTTTTCTAAATAACAATTTAT
AATCGTGTAGTAAATTGAATGTTTAATCATAATATTTGTTAGGAATTAAAATCTTTATTTTTAATGGCAGT
CTATTTCTTATTATGATCCTTATTTATCTGTATTTTTACTGTCAGGTTTAATTTTGAAAAGATTTAAAACAT

CTTGTCATTATTAAAATTTTTCTTTTCATTACACTTATTCTAAGAATAGAATATAAAATGCTTTATTGAATG
GTTGCCTAAAGCCGCCATCAGATTGATACAATTTTAATCGTTTAATCAATGGACTGGTTGGTAGGATACT
GAACTTTTTTTATTGATTTGTCTGGAACGTTTTAACAGATGGCCCAGCTGTAGTTAATATTAACCTGTT
CGTGAGAAGTATTACGACTATAAGCGACATTAAGATGGTTAGTAAAGAGACAGAGAACAATGTTTCCT
TTTATGTTAGTGTCGTTAGTAAAAGAGAGTATGAGCCTTTAGACAAGACTTCAATCATCGATCAAGAAA
TCGGAATCTTTGAGGCGTGAGGTCAACGTACCTTTTCTTTCGGACGGTAATATCATTTATAAAATGTCA
ATCGATTACAATTGTGACATTTCTATTTCTTGACGATCTCCGATTGTCATCTTGTGTGAGTGGGCACACC
GTAAAGAGGTTGTACCGTTTGTTTGTTTAAAGAAAAAACAGTTGAAAATTTTCGAGATTTTTCTCCATG
GAAAAAATTGTGCGTTTAATTATCCAAGCTCGTACCTATATCTTGCACTTTTTGAAATATATAGGAAAAT
TTAGTTAGGCGGCAACGTTTGTTTTAGTTTCATAAGACCTTATTTAGGCAGATTGGCACGTACATTTTAC
TTAGAGTTTGTATTTCTATTCAAAATTTTTAAATTTTATTTTTGTGTTTCCTATTGGTAATTTTCGTTTGTT
CGTATAGCACGTACATTTTGCTCATAATTACGATCTTGGACAAATTAATATTGAATATGAAACGTATGAA
AACATGAGAAACAAATGTTACAGCCTATTTAACTGTTTTCGTACGGGAGCGGGCGTGTTACGGTAAAC
GAGAGAGCAGTTCCTTTATTATGTTTTTCTATTAAAATAATTAAATTATCTATAGTATGATGACATCATCTG
CTGTCTTTTTATTTTGCTGGTGATTTTCTTGGTGATTCCAATTGAATTCCTCGTTATTATTAATATTTTGTT
TAGTTATTAATGTGTTTTAATTTCGTTTTTTATTTAATTTTATTCTTAAGTTTTGTTTGAACTTGATAAGGT
GTTTTGCAGCGTGTGTTTATCTACAGGTTATTTTAAAAAAAAAGGTTGACCTCAGAATTTTATTGTTATC
TGTATTTGTAAAAAAAAATGTACCGCCTTCGAAATGTGACATATGATTGACATAAACACAATTCTATTTTT
AAATTAAGACGCTGTAAACAAACGGAACGGCTGATTGAATTATTGTAAAAAGTTAAAGCATTTTCCAA
ACATTTCCGTACTTTTAGGAAACAAATATTTTTAGGACAAAAACATTATCGTTAGGATATACTTTTTACA
CTTTCCGTGTCACTATAATAAATAGAATTAGTCGATGGAACATTGCAGTATCATTAACTATAATGGATACT
ATATTTTATCGACATCACGCGATTGGTACAATTTCTTTGAAAAACAAATAAATAATCACGATATGCATTT
GTTGTATAGAATTAGATATTTGTTTTAAAATTGCTGTCCCATTTTCATAAATTGCAGTTCATTTATCGATTG
AAGATAATCTTTTAACACGGAATAATTGAAGTATAGGTTATTACAGATATTTATGTTGAATTTTAAAACG
ACTGTCAATGGGTAAATAAGAATTGAAACCGATAGCATCGTTATAAAGCTATTAGGGGCAGCGTTTTAT
TATTTACTCCGTTAAAACCATTCCAATGCTTTCCCCGATGTTATCATCTTTACTTAGATGGTTTAAATAGT
GGGACAAAATGGCGACTTTTATTTTTATCCAATCGTAGTTAGGTTAGAGATTCTTACTGACACACTTAG
AGCCGTTTAATGATTACTTAAATTATTCCTTAGTAACGTTTTTGTATCGAAAACAATTGCTAAGGACTGG
TTTAAGTAACCATTAAATGACTTTGAATGCGGCAGTTAGACATTGATTAACTCAGTTTGGGTCAAGAGG
TTTTTTTGGACCATTTAATAGTTCATACGATGTTTTAATTGAAATTAATTCAAGTCCTATTAGTAATGAGC
TGTCGATAAATCTAAAAAAACAAACCAATATTTATTTACAAATTGTAATTTGCACGGAAATAAATTTAAA
GTAGATGTCGCTTCTAAACCCATAAATAAATTAAACGGTAAATTCAATCTGACAGCCAACTTAATCCGG
ATTAATGTGAAACTAAACAAATGCTATTTAGCAAGAAACACGTAGCTTTCTAAAACAAAATGTTAATTT
CCGTTACACGCTACCTTTCAACCCAAGACGGTGGTATCTCGACCCTCGATCGTGATATTGCGTTTTTGT
ATCGACATCACGGAATATGTACTTACTTAACGAAAATAATTATTCTTTAATACGAAATTGTAGCCATTTTT
TAGGTCATTAATAATTACCTATATTTTAATACGAAAATGTAGTCACGTTTTAGGTCGTTTAATAAAAAACT
AAATGAAAAAGTCCGCCATATTTATTGACGGTTAATTCCGTTTCTTATTTCAAAATTTTAAATTACTGTC
AAATTAATGTCATGTTAGATTTTTTTATATTACATAGGTATATAAACCAGCCCTCTAAAGCCACGAGAATT
GAAATTGAATAGTATCTTTACTTAAATTTTAGTTTTCTTTTATATTTTATGACTAGTTTATGTTTTTGTTTG
GTTTCCAATCTGATAGATACTGCTCTATATTACACTAACACAAATTCTCGTTGACAAGCCGACAGTTGA
GATTGTTTGCAGCGTTTTATTTTGAACCGATGTCGTTTCATTTGATGTTAGTGGTTTGTTTGATCACAATT
ACATTTATGTTGTTTGTGTGATCAGTATGTCGGATCATTCATGAGCATAGTGTACCCACATTAATGTTTAC
AGGAGACAGGTGAGTCGCTAGTCCTACTGATAATGATAACGCGCATTCACAAACTAATGTTAGGCCGT
AGTATCATAATAATACCGTGATTTATCGTATATTTTGCTTGGTACTTAGATGGGCCAGCGGTAGTGAGCGT

CAATATATTTGTCCGAAGTATATCAAAGATCGATGACGTCACAATGGTAAGTCAATGAGGACAGACTGG
GCCGCAACTAGTGTTATTACCAAAGTATAAGTTTTCTGTACTCACGCGGTTCTAACAAGTCAAATGAAT
GGGCAACTCTCTCCTGTTGAGTAGGAGTGATTTACCCGAACACTATGTGTAATGTTCATTTTTTTCTTAT
TCTTTATTTTTTCCTTTCGATTTCTCTTTAATCCCGTTTGGTGTTGACGACCAAAATCGTTTTTAGCCGCG
CGTCTGTTACTTCTTAGAACGCCAGTACTGGTATAATCCTAGCTTTGTTCATCATCTAAAACTTACTATAA
ATAGAATAAAAAAATAGTCTTTATAGTATTTAGTTTTGTTTATTTTAATTTAGACTGGCTGGTTGTCCTTA
CGAGTTCATCTAACGGGGCATAAGCTTTCTGTAATGAAGTGGAGCGCGCGGGGCGGGCGGCGGTGGC
GGTGGTGGCCGCGGCGGCCGCTCCACTTCGTTATATCCTTTAAGCATCTGACATTATCATCAACTGCGT
ACTTCTCTCGCGTTGCTCTAGTGGTTAGGCACTATTTGTATGAAGCGGTACAAACAAAATAAAATTTAA
ATTGTTGTTTTCTGTCGTTAAGGCTATGCGCCAACGTTAGTCCATGTCAACATGTATCTACGGTCCATCA
GCAAAATAGATGATTACAAAATGGTGAGTGTCTTCACATAACCTGATGTAATCCGTGACATTTTTATTAT
AAACTAAAGTTAGATTTCTCTGTGGGATTTCGCGATCCATTGCTCTACCGCCGGTGCAACCCAATAGTG
AGACAGTAACCGCGGCAGCAGTGGACCGCGCCCGTCTATTAGCTCAGTCTTGCCAACTTTGTTTATAAT
AAAAATATAAATATTATGCAATATCTGCACTACGCTAAACATTCTCAAGCTATTCGTAAATAATTGTGTTC
GTGTATTTTGTTAATTTTGTTCTTTCCCCTTTAATTTTAGTTGGTTTAATATCAGTTGTGCACCTTTTCTTT
CCTTTTCCTAAATGTATCATTTAACCACCCTTAATGTAAAAATTTGAATGTTTAACTAAATATTTCGCTAA
ATAAATCCCGTGGTGCGGTGCCGCGCACTTTATTTTTATTTTTGTTTTTATTTTATTATAAACTCAGACTA
AGTGTAGTCTGGCAACTAAATTATAAATAATGTTTTAATTTTAAATCTTGATCAAAATTACAGCTTTTAAT
TTTCTTCGTGGAATTAGATCTTTTCTGTTTCTTTACTTTCTCTTCCTTATTATTTATATATTATGTATTTGTT
GACTAGCTACACTCCTGCAGGCGCGACTCGAGCTGTACAGACACGTGCAGGATGGACTATTGGCACTC
AAGTAGTTAGCACTCAACTGTTGAGCAGCAAACTTACCGCTTCGGTATCACAGCACTGATACGTTCAA
AATCAATTTGGACTCTATTGCCTTAGAAATAAAGAGGAATCACTTAAGTTGTAAAGTGAAGTAATTCGG
CCACGCTTGTAGGAACCACGGGGACCGCGGCCCGCACCAGACACCATCATTTTATTTATGTTTCTTATG
TTTATTTGATCATGCTTCTTATGTTAAGTTCACGCAGATGAGGAAATTATAAAAACTATTTTGGTCGTGT
ACGTTGATTGTAATGAATTGTTGATATGTCTTGTTCATGTGAATTTGTGTTGTGTAATCGGGACTTACCG
CCGCGCCCGCACTCGCCTTCGTGCTTGGAACGGTGGCTGCCCACCAATATGTGACACCCGACCCCAAA
CAACAGCTAGACGTACCGTAGCTCTAGTTATACTGGCCGATTGTGCATGAAGGTAATAAAAGCATTCCT
TCAACGAATGCTGGAGACGGTAAGTTCGAACTGGGCTTCGCTTCTGTGCATGTTGCTTCGAACTATGG
CGCTCGAATCTTCACTTTTGGATGGCGATGATTTATTGAGCCAGTGGAGCTGACAGTAGCGGGAGCCTT
CATTTCCTTCACGACAAGTACCCAGTTGCCCACATGTACAGATGTATATTGTATACCGTAAGTCCATCAA
GTTGCCCTCCATCATTACCGCGTTCGTTTGTTTCGTGTCCAAAAGTAAATGTTCGAGTGTGCATGTCTG
ATCTTCTTGTTGGAGCATCTGCCGGCTTTTGTTGTACGATAATTCATTTGACTTGCTGAGATGTCGAGGG
ATCCTCTAGTGCATTGTGCCTTGATGTCAAATTAATTATTGAGCGAACCTTCGACACCGTCCCCTCCCTT
AGTTGTAATATTACATGTAAATAAATCATTTTGTGCCAGTACCGCCTCGTTGGGAACTTGCACCTAGTAC
TCTCGGCACCTCTTTGTACATTATTGTATGAAAAAGTTACTAAAAAGGACGATATTGGCACAAATAAAG
ACATATTAAAAGCTTACTGGTAAAATAAAGTCATTAGTGAATAGCTTTGTGCGATAACTAAAAGACTAG
ACGAATAAAATGGTTTTAACAAAAGACGAAGCTTTTAGATGCACAGATGCTACGAAAGCAAAGTGGA
GAAATGTCTTTAGTATAGAGTAATAAATGGGGTCTACATTGAAATATTGTCAAAATAATTAATTAATCAC
GTCAACGACAAAAGATGTTTCGTTAAAAACGGTTGTCACGAAAATCGTTATGTAGATTTATTTTACTAA
AACCTAATGTAACGATGAATATCGTTATTTATATTAATCAGGGTTTGTTGGAGTTTATACAAAGTGTTCA
GACTAATGATGAGTAACAGTGTGTTCTTCTTTGACAATATATCAATGTTTTTGTTGGGATATTGACAACT
GCTACAAATAATGTCATGTTTTCAGGAATACTCGGTACAGTTAACGTTCCGAGAACAGTGGTTAGATGA
GCGCCTCAAGTTCAATAATTTGGGAG

Text S3 The *SfGluCl* exon 2-4 sequence obtained by experimental sequencing

Note: Exon sequences are shown in gray; the rest represent intronic sequences.

ATGTATGAACGGTGGGAAGATAAATTTCCGAGAGAAAGAGAAGCAGATCCTCGATCAGATCCTGGGTC
CTGGGCGTTACGACGCCAGGATCCGACCTTCAGGCATCAATGGCACTGGTAAGTCCTCGCTTCGCTTA
CAGCTTCATTCGGTCCGTGGTGGAAGCGATAGCTTAGGGTCTAGGGAGATAGGATGGTCTAGGGAGAT
AGGAAGAGTAGATGGACAGGTCTAAGCAAAACTGTGGCAGACAAATTTTAGCAGGTCAAAGAGGATA
GGTAGTGAATCTGGTCAGAACTTTAACATAAATGGAAAAAGACCAGGAGAAAAAAGTAGAATGGATG
ATTCAACAGATATTTTTAATACATTTTATAGTTACTAACGAGGTCGAGTGGTCGCAAGTGCGACTGCTG
GACAAGGGGGTCTCGGGTTTGATTCGGGTTGGGCAAAGCATTACTGAGCTTTTTTCGGTTTTTCGAAA
ATTTCTCAGTATTAGCACGGAGTCTGAAGTTGTGTCCAGTATATGGCAATGGGCTCACCCTCTATTACAT
GGGGACTTATAACACAAATTGAGAAAAGTGGGTGTACATTGCAAATAGCGGCATTACGTGCCGTAATG
TGCATCTCTGCCTACCCCTTCGGGGATAAAAGACGTGACGTTGTTATAGTTTTATAGTTACAAATCTTTG
TTTAGGAATCATGAAATAACAAAACATTACAGCTAAATGTCTTTAATGTGAAATCATAATCGTAAAATGT
GAGAACACAATAAACTTGATTAATGTTGTAAATCATGAAGTAATAATAGACACAGACACATTTATTTTG
ACACACATCTGTTCAAAACATGTGTTGTCTTGGCTATCAGCTATTTATGGAACTAAAATATATTTTAGTT
GCCGGCCATTATTAATAGAGTTAGCTCTTATATTTGTCTCGTTCGAAAATAACGGTCGAATTGATTATTGG
CGGTCATTTTTTATTTTGGAGTCTTTGGCGAAGATTTGTTGTCGTAGGACGATGAAGTAAAAAGGACTT
TAGTGAGGAATCGGTTTTTGACACTGTAATGTGTTTTATTTTCAATGACAACTGCACGGTTGGCGCGGT
GGCTGGGCAACTGGCTGCCGCACAATGGGTAGCGAGTTCGTTTCGGACCAACTCTTTGTGTCATCCAC
AAATTATTATTGTTTCGGATCTGGATGTCATGTGTATGTGAACTTGTATGTTAGTCAACGCACCCATGAC
ACTGGTTAAAATCCTAGTGTAAAACATAAAAGGATTTGTCTTTTACAACTTAACAAAACACCTGACCA
AAAATAAACGAGATCCTCTTACAACCTCTTGAAAAACTAAGGTCAACTAAAAATAAGTTAAAGAAACC
CACTGACCTTACAATTAAATACTAAAAACGTATTTTTGTATAAAGGAATGATGATAAATGTATTTTTTATT
TAGAAGCACTTAGTGCTAAGATCTGTACCTTTGTAAGGAAGATAGAGAATGTTGAGAATTCTAAGGGT
CTAACAAAAGACGGCCGCCATCTTGGAATTGCCACGACAATCATTTATCGTCGGTTTTCATGCCAACTG
TTGGCACCTTCTGACAATTTTCTTCGAGATAAATAATAACACTGTTATGTCTCTAAGTTTGCCTTTTGAA
ATGTTTCCCTTTTGTCTCCATGTACTGATTCTAGATGTTTTAATGTTTGTTTGTAAGCTTTTTTTGACGGG
AAAAGTGTTTGCTATACATTCAGTTTTTCTAAAACCTTACTGCCTCACACAGTAAAACTATGGAATGTT
CTGTGGTCGCCGGTATTTCCGAACCAATTCCTTCAAACATTCAAGAAAAGAGCGTATTTCTTTTTAAAA
GGCCGGCAACGCACCTGTATCTCCTTTGGTGTTGTAGGTGTCCATAGGCGGATCTAATCGCTTACCGTC
AGGTGAATAGAAAGCTTGTTTGCCTCCTATTCCATTGAAAAAATCTTTTACCTATCTCTCCGTGATTCAC
ATTATTAAGCAGTCGTATCTCGAACATAAGAAAAATCTCTAAACATATACCTCAATAGATTGAATAGCAA
CAAAATCCTATATTTACCGACACACAAAACGTAATCCCTTCAGACCTACCACATCGTCTGCCAAGTCAA
ATTCGCATGCATGACAAACGCGTCTTCTTAATGGTTCCGTAATGTTTGTGTAACAACGTTGTCTGTCCAT
TAGTCACTATTTACTTCACCTGCTCTTCTCCCTTACATATATAATATACTATGTTCTGCAAGCGACTTCAC
TCGCATTCCCATGACATAAAATATAGCCTATGTGTTATTCCATACCATTAATCTACCCCTGTTTTCCGTAG
CTGCGGACTACCTAGCCGCTTTACTGGGGCTCCGGCTCGGAAAGCAGGAGTAGGAACAGGGTGGTTT
TAGTCAGTAAGAGCCTGATACTCCTTCTCATCTCGTTCAATGCGGGAAAAGTCATTCGTTGATAATCTC
CCTATAAAAAAACCCCGATTTCAGCCCGATTCCTTCAACTGTATAGACGTTTTTGGCTAAAAATATATAC
ACAAACTTACTCATAAACTTTATTAGTAAGATGGCAAAGCTTTCTACATATTATATGCTTTGTACATCACC
CCTACATTTGGCACTAACATCGGCGGATGTGTTTAATAGGTTCTTGGCATTTGTACATCTGATTCAGGTG
CTCTCTCATCATAGTTTATTAGCCCTTTCAAGTCAGACAGTAATTTGGTAGTTTGTTATTGCAATATGATT
GGGTATCTGGCTACAGATGTTACTCTGTCTACGCTCTTAATAATATAAGATTTTGAAAATGTCTGTTGAA

GTAATCACTGTTAATGAAATGCTGTATAATTTACACTTATATTTAAAGTTTATTTTTTGATTTAGTGGTATA
GGAGAAAAGAACTGTAATATATTATCTAGAATCAAGAATATCTTTAGGTATCTGAAAAAGCTATCGCTTC
CACGACATTAAGACCACAGCTAATTCTAGTTACTTAGAAAACACTAACTGTCAACGTTATGATATTAAC
AAACCGGTAAACTTGCTGATTGAATTATAATTTTGTTTTCAAACTGAAAAGTAGAAACAGTCGCTCACG
ATGTATATAAATATCAACTTTTTTCTTTTATTTTATGTAAATGACTATGCTATTTTTAATTTTGTTCGAATAG
TTTTCTCAAAAAACTTTAAGGTCGCAAAAAATAATACTTTCTTAACTAACATATAAATAAAATTCGCAA
ACCAACTGGCTCTTTAGTTACAGAGATAATAAAAAAGAAATAAAAAGTCACCGCGGTGCGTTTATGTG
AAAGATTTAAAAGCAACGCTTTGCCAAATTCTGCATCGATAACATTTACTTACAAAATGTATTGCAATG
AATATATTAGAGACTGGAATGTGCATACCACATGTACACACAAGTACCTACCCACGGCGGGAAGAGAA
CGCGACTTAAAATAAACTACGGATTGTGCGCAAACTGCTGGTGTAGGCTGAATGTCGTAATCCGAGTA
ATTTGTTACGTTTTTGTTGTCTTCTGTTAATAAAAGTTCAATTACTGCAGCCAATGTTTCCTCCGGTCAA
TACTTTTGGCGTCACTCCAAATATTTTCTAGTTACATATTTAAAACTGTCAAACGATTTATTTGGTATGCG
ATCTTTGCCCTTTCTGACTTTCTTATTGATTTGTTTCAAATATATAGTTATATTATTATTATTCTCAAACAA
GATATGTTATATTCATGAATTCTTTTCATCTTCGTCAATGTGAAATAGTGTATCTGTTTATTCATTTATTCT
GTAGGTCACATCCAGTAGGTCGTGCTCACCTCTTTTAATATCTATGTCAAAAATCCGTCTTTTTCGTATT
GTGTATTGAAGCGTAGTCATTTTTCTATAGTGTGTGTGTTGTTTGAAAGGTTTTTATCTAATATCTTGGCT
ATTAAAGGGAAGTCGTTTGTCTGGCTGTCGGAACACATTAGCTTGATTCTTGATATTGTGGGGAAAAAC
ATCGTAACGTTATTTGTACTCGTACCTACCTACCTATGTATGTGTATTTTGTTCCTCATAGGTGCGATCGA
TTTCTTTACGAAGACGTTTCAGATTATGTTATTATTTCAGAATGAAGATTTGTATAAAATATTGTTTGCTT
GCTAATAATTTTTCACAGGAAGCTAGGCATAATAAAACCACAGGCACCACTATTATTATTTCCTGTGGCC
ATAGATTACATCACGCTGTTCATTATCTAGAATATATTAAGATATCTCAACGAGATTGGTTTTCTCGAGAG
ATACACAAAAAAGCATCGGAGTGCCACTCAAGTGCTACATCTCGTAATTTCCACAAGTTCATTGCGTAA
CAAAAGAGGAGTGTTACGTGAAAACCATAAATCACGCCGCGATTTTTTTCCGCCATCTTTATTTTTACA
ACTTTGCGCGGGAACGCGGGAATCTGTAATGGAAGAACATTTCACGGAGCCTCCCTGAGCGTGATTTT
TATGATTCTATACAGTTTTCTCGTTAATGGAAATGGCATTTACACTTTAAGAGGTTCGTGGAAATGTGCA
AGTCAGAGGAGAGGCTTGTAACCTCTTTGCTTCCCTTTATTTAGATATCTATTGGCTGAGAGATGAAGG
CGAGTCTTTAGGGATACAAATAATTCAACTTAACGTGTTATTTGACTGGTTCCGTCGCATTTGTCATCAT
CACGCTACTGACTTTACCACCTTCCTAGATACTGTTCCTTCAATGAAATCATAGCTATCCATAAATCATG
AAACTCGATTTACCCATTAAACTTGGACTTCAACCATTTTATATACAAATGTGAGAGAAGTTATTCATCA
CTTAAGCTCTGAATGCGAGATTGGATAAGTGAGGTGGAGCGCAAAATGACACCATCCTCCTTGATGAG
ATAAGAATAAGAATTGTAAAGAAATTGCAGGAAATTAATAGCAATCCTCCTTGTTATGTTGTTAGTAATC
GTTTGAGTGCTACGTTACATATTATTATAAAAGCAATAAACTCACATTGTAAAAGCGAATTTTATGAGAT
GTACTTAGCTATGTTGCTCTTTAATTTTGATGTTTGGAGTTGTAAAAAGTAAGATAGTTCTATGTAAAAA
TAAACAAAGATCTATCCATAGAAACAGGATTTGTTATTCCCTCTTGATAACTTATTACTGACTAGTTTCA
ATAATTAAAAAACAAAGAGTAGAAGGACTATTCGTATATACGCAACGTAACGCCACACATTTTTATCAT
TGAAGGAGTAGGCAGAGGTGGACATTACATCAAGTAATGCCACTGTACAATGTACACCCACTTTTCAC
TATTTATATTATAAATCCCATGTAATAGACTTGAGCCTATTGCCATATACTGGGCATAATTCCATACTGCTA
TAACTAAATACAAATCTCATCAACATATACAGCCAAATTGTTACCAGGTACATTTCTACGCTAATTATTTC
CTCAACAATGTCCGCGATAAAATTAATCACATACGTTCCAAGTTTCTTTTCATTCATTGTCGCCGACGTC
TGCCACCATTGAATTGACCCCAAAAGGTTCCACAAAGGTTCCATTAATCACCCTTCTAATGAGCCAGG
GTTGCTGCTTTTCTTAATCAATTATTCATGCTCTAAAAATATCAGGTCCAGCTCGCTTCAGATTAACCATT
CGTTGCTTTCAGAGTCTTTGAAATACATAGATATTAAAAAGACTGTTGTTTTCTGTTTTCGTACAATTCG
CGAGTGTTGATGAAAAGGTTATTTATTTTCTTTTCATGTTTTTTGAGGTTATTTTTTGCTAAATGTGTTTT
TGTAAGGTTGTGTAAGGAAGTGTGCTTTTGAATGTTGTTTAGTGGGAAATTATGTAGGAATGTCGTGTC

ATGACCAAAAAACATCGCCTTGTGATCAGTCTTTGAAGTTATATCCACAAAAGTTAAGGATTTTTCTCA
TTTGTAACGGTTTTTCTAGTAAGTTCTTTGTCTAAAGGTAAAATGTATTTATAGGGAAGCAATAACAGA
GTGTAGTTAGTCGCCAGCCCTGTTTATAAACATTCTATTTGCGGTGCTCGGCTGTCAAGTACACTGTATA
CCGTATTGAACCATTTACAAGCAACTCACTTCTTTCTCCTTTGAAATGGCTTTATAAATAGTTTACCTTTT
ATTACACTTATATTACAAATAGTTTATTGTTCATTAAACCTCTATTCTATATTGAAACTCAATATCAATCAT
TAATAATTGAACAAAGCATTATATTCTTTTTGTTTTCTCTCAAACTCTAGCCTATAATTAGGCTGGAAATT
TCTAGTAGCTGTTTTCGATAAATTCATTGATTTCTAACCTACTATTAAACCTTATAGGTTTTTTACCATTAT
TATCCTTTAGAAGCGAGTAACAATATTAATAATAAACTATTAATTTCCCTTTGGCTACCTATTTACGTGAA
TAAACGTTGAATTATTGATGTTAACTTACACGCAAACGCGAGCGTTAACCGTTTCCCAACACATTAGAT
TAAACTCAAATCGGTTAAACCTAGACATGTTTGGGAGCTATCGTTTTTCTAAATAACAATTTATAATCGT
GTAGTAAATTGAATGTTTAATCATAATATTTGTTAGGAATTAAAATCTTTATTTTTAATGGCAGTCTATTTC
TTATTATGATATTTATTCTATCTGTATTCTTACTGTCAGGTTTACGTTGAAAAAGATTAAAAACATAAATC
TTGTCATTATTTAAATTTTTCTTTTCATTACACTTATTCTAAGAATAGAATATAAAATGCTTTATTGAATGG
TTGCCTAAAGCCGCCATCAGATTGATACAATTTTAATCGTTTAATCAATGGACTGGTTGGTAGGATACTG
AACTTTTTTTTTTATTGATTTGTCTGGAACGTTTTAACAGATGGCCCAGCTGTAGTTAATATTAACCTGTT
CGTGAGAAGTATTACGACTATAAGCGACATTAAGATGGTTAGTAAAGAGACAGAGAACAATGTTTCCT
TTTATGTTAGTGTCGTTAGTAAAAGAGAGTATGAGCCTTTAGACAAGACTTCAATCATCGATCAAGAAA
TCGGAATCTTTGAGGCGTGAGGTCAACGTACCTTTTCTTTCGGACGGTAATATCATTTATAAAATGTCA
ATCGATTACAATTCTGACATTTCTATTTCTTGACGATCTCCGATTGTCATCTTGTGTGAGTGGGCACACC
GTAAAGAGGTTGTACCGTTTGTTTGTTTAAAGAAAACAATTGAAAATTTTCGAGATTTTTCTCCATGGA
AAAATTATTGTGCGTTTAATTATCCAAGCTCGTACCTATATCTTGCACTTTTTGAAATATATAGGAAAATT
TAGTTAGGCGGCAACGTTTGTTTTAGTTTCATAAGACCTTATTTAGGCAGATTGGCACGTACATTTTACT
TAGAGTTTGTATTTTCTATTCAAAATTTTTCAATTTTATTTTTGTGTTTCCTATTGGTAATTTTCGTTTGTT
CGTATAGCACGTACATTTTGCTCATAATTACGATCTTGGACAAATTAATATTGAATATGAAACGTATGAA
AACATGAGAAACAAATGTTACAGCCTATTTAACTGTTTTCGTACGGGAGCGGGCGTGTTACGGTAAAC
GAGAGAGCAGTTCCTTTATTATGTTTTTCTATTAAAATAATTAAATTATCTATAGTATGATGACATCATCTG
CTGTCTTTTTATTTTGCTGGTGATTTTCTTGGTGATTCCAATTGAATTCCTCGTTATTATTAATATTTTGTT
TGGTTATTAATGTGTTTTATTTTGTTTTTTATTTAATTTTATTCTTAAGTTTTGTTTGGACTTGATAAGGTG
TTTTGCAGCGTGTGTTTATCTACAGGTTATTTAAAAAAAAAAGGTTGACCTCAGAATTTTATTGTTATCTG
TATTTGTAAAAAAAAATGTACCGCCTTCGAAATGTGACATATGATTGACATAAACACAGTTCTATTTTTAA
ATTAAGACGCTGTAAACAAACGGAACGGCTGATTGAATTATTGTAAAAAGTTAAAGCATTTTCCAAAC
GTTTTTCGTACTTTTAGGAAACAAATATTTTTAGGACAAAAACATTATCGTTAGGATATACTTTTTACAC
TTTCCGTGTCACTATAATAAATAGAATTAGTCGATGGAACATTGCAGTATCATTAACTATAATGGATACTA
TAGATATTTTATCGACATCACGCGATTGGTACAATTTCGTTGAAAAACAAATAAATAATCACGATATGCA
TTTGTTGTATAGAATTAGATATTTGTTTTAAAATTGCTGCCCCATTTTCATAAATTGCAGTTCATTTATCG
ATTGAAGATAATCTTTTAACACGGAACAATTGAAGTATAGGTAATTTTAGATATTTATGTAGAATTTTAAA
ACGACTGTCAATGGGTAAATAAGAATTGAAACCGATAGCATCGTTATAAAGCTATTAGGGGCAGCGTTT
TATTATTTACTCCGTTAAAACCATTCCAATGCTTTCCCCGATGTTATCATCCTTACTTAGATGGTTTAAAT
AGTGGGACAAAATGGCGATTTTTATTTTTATCCAGTCGTAGTTAGGATAGAAATTCTTACTGCCACACTC
AGTGTCATTTGATGATTACTTAAATTTTTCCTTAGTAACGTTTTTGTATCAAAAACCATTGCTAAGGACT
GGTTTAAGTAACCATTAAATGGCTTTTAATGCGGCAGTTAGACATTGATTAACTCAGTTTAGGTCAAGA
GGTATTTTTTGGACCATTTAATAGTTCATACCGATGTTTAATTGAAATTAGTTCAAGCCCTATTTGAACTG
AGCTGTCGATTAATCTAAAAAATAAATTAAAACCTTTTTAATTTGCACGGAAATAAATTTAAAGTAGATG
TCGCTTCTAAACCCATAAATAAATTAAACGGTAAATTCAATCTGACAGCCAACTTAATCCGGATTAATGT

GAAACTAAACAAACGCTATTTAGCAAGAAACACGTAGCTTTCTAAAACAAAATGTTAATTTCCGTTAC
ACGCTACCTTTCAACCCAAGACGGTGGTATCTCGACCCTCGATCGTGATATTGCGTTTTTGTATCGACAT
CACGGAATATGTACTTACTTAACGAAAATAATTATACTTTAATACGAAAATGTAGCCATTTTTTAGGTCAT
TAATAATTGCCTATATTTTAATACGAAAATGTAGCTATTTTTTGGGTCGTTTAATAAAAAACAAAATGAA
AAATCCGCCATGATTATTGACGGTTAATTCCGTTTCTTATTTCAAAATTTTAAATTACTGTCAGATTAATG
TCATGTTAGTTTGTTTTTTATATTACATAGGTATATAAACCAGCCCTCTAAAGCCACGAGAATTGAAATT
GAATAGCATCTTTACTTAAATTTTAGTTTTCTTTTATATTTTATGACTAGTTTATGTTTTGTTTGGTTTCCA
ATCTGATAGATACTGCTCTATATTACACTAACACAAATTCTCGTTGACAAGCCGACAGTTGAGATTGTTT
GCAGCGTTTTATTTTGAACCGATGTCGTTTCATTTGATGTTAGTGGTTTGTTTGATCACAATTACATTTAT
GTTGTTTGTGTGATCAGTATGTCGGATCATTCATGAGCATAGTGTACCCACATTAATGTTTACAGGAGAC
AGGTGAGTCGCTAGTCCTACTGATAATGATAACGCGCATTCACAAACTAATGTTAGGCCGTAGTATCAT
AATAATACCGTGATTTATCGTATATTTTGCTTGGTACTTAGATGGGCCAGCGGTAGTGAGCGTCAATATAT
TTGTCCGAAGTATATCAAAGATCGATGACGTCACAATGGTAAGTCAATGAGGACAGACTGGGCCGCAA
CTAGTGTTATTACCAAAGTATAAGTTTTCTGTACTCACGCGGTTCTAACAAGTCAAATGAATGGGCAAC
TCTCTCCTGTTGAGTAGGAGTGATTTACCCGAACACTATGTGTAATGTTCATTTTTTTCTTATTCTTTATT
TTTTCCTTTCGATTTCTCTTTAATCCCGTTTGGTGTTGACGACCAAAATCGTTTTTAGCCGCGCGTCTGT
TACTTCTTAGAACGCCAGTACTGGTATAATCCTAGCTTTGTTCATCATCTAAAACTTACTATAAATAGAAT
AAAAAAATAGTCTTTATAGTATTTAGTTTTGTTTATTTTAATTTAGACTGGCTGGTTGTCCTTACGAGTTC
ATCTAACGGGGCATAAGCTTTCTGTAATGAAGTGGAGCGCGCGGGGCGGCGGTGGTGGCGGTGGTGG
CCGCGGCGGCCGCTCCACTTCGTTATATCCTTTAAGCATCTGACATTATCATCAACTGCGTACTTCTCTC
GCGTTGCTCTAGTGGTTAGGCACTATTTGTATGAAGCGGTACAAACAAAATAAAATTTAAATTGTTGTT
TTCTGTCGTTAAGGCTATGCGCCAACGTTAGTCCATGTCAACATGTATCTACGGTCCATCAGCAAAATA
GATGATTACAAAATGGTGAGTGTCTTCACATAACCTGATGTAATCCGTGACATTTTTATTATAAACTAAA
GTTAGATTTCTCTGTGGGATTTCGCGATCCATTGCTCTACCGTCGGTGCAACCCAATAGTGAGACAGTA
ACCGCGGCAGCAGTGGACCGCGCCCGTCTATTAGCTCAGTCTTGCCAACTTTGTTTATAATAAAAATAT
AAATATTATGCAATATCTGCACTACGCTAAACATTCTCAAGCTATTCGTAAATAATTGTGTTCGTGTATTT
TGTTAATTTTGTTCTTTCCCCTTTAATTTTAGTTGGTTTAATATCAGTTGTGCACCTTTTCTTTCCTTTTCC
TAAATGTATCATTTAACCACCCTTAATGTAAAAATTTGAATGTTTAACTAAATATTTCGCTAAATAAATCC
CGTGGTGCGGTGCCGCGCACTTTATTTTTATTTTTGTTTTTATTTTATTATAAACGCAGACTAAGTGTAGT
CTGGCAACTAAATTATAAATAATGTTTTAATTTTAAATCTTGATCAAAATTACAGCTTTTAATTTTCTTCG
TGGAATTAGATCTGTTCTGTTTCTTTACTTTCTCTTCCTTATTATTTATATATTATGTATTTGTTGACTAGCT
ACACTCCTGCAGGCGCGACTCGAGCTGTACAGACACGTGCAGGATGGACTATTGGCACTCAAGTAGTT
AGCACTCAACTGTTGAGCAGCAAACTTACCGCTTCGGTATCACAGCACTGATACGTTCAAAATCAATT
TGGACTCTATTGCCTTAGAAATAAAGAGGAATCACTTAAGTTGTAAAGTGAAGTAATTCGGCCACGCTT
GTAGGAACCACGGGGACCGCGGCCCGCACCAGACACCATCATTTTATTTATTTTCTTATGTTTATTTGAT
CATGCTTCTTATGTTAAGTTCACGCAGATGAGGAAATTATAAAAACTATTTTGGTCGTGTACGTTGATTG
TAATGAATTGTTGATATGTCTTGTTCATGTGAATTTGTGTTGTGTAATCGGGACTTACCGCCGCGCCCGC
ACTCGCCTTCGTGCTTGGAACGGTGGCTGCCCACCAATATGTGACACCCGACCCCAAACAACAGCTAG
ACGTACCGTAGCTCTAGTTATACTGGCCGATTGTGCATGAAGGTAATAAAAGCATTCCTTCAACGAATG
CTGGAGACGGTAAGTTCGAACTGGGCTTCGCTTCTGTGCATGTTGCTTCGAACTATGGCGCTCGAATCT
TCACTTTTGGATGGCGTGATTTATTGAGCCAGTGGAGCCGACAGTAGCGGGAGCCTTCATTTCCTTCAC
GACAAGTACCCACGTTGCCCACATGTACAGATGTATACCGTAAGTCCATCAAGTTGCCCTCCATCATTA
CCGCGTTCGTTTGTTTCGTGTCCAAAAGTAAATGTTCGAGTGTGCATGTCTGATCTTCTCGTTGGAGCA
TCTGCCGGCTTTTGTTGTACGATAATTCATTTGACTTGCTGAGATGTCGAGGGATCCTCTAGTGCATTGT

GCCTTGATGTCAAATTAATTATTGAGCGAACCTTCGACACCGTCCCCTCCCTTAGTTGTAATATTACATG
TAAATAAATCATTTTGTGCCAGTACCGCCTCGTTGGGAACTTGCACCTAGTACTCTCGGCACCTCTTTG
TACATTATTTGTATAAAAAAGTTACTAAAAAGGACGATATTGGCACAAATAAAGACATATTAAAAGCTT
ACTGGTAAAATAAAGTCATTAGTGAATAGCTTTGTGCGATAACTAAAAGACTAGACGAATAAAATGGTT
TTAACAAAGACGAAGCTTTTAGATGCACAGATGCTACGAAAGCAAAGTGGAGAAATGTCTTTAGTATA
GAGTAATAAATGGGGTCTACATTGAAATATTGTCAAAATAATTAATTAATCACGTCAACGACAAAAGAT
GTTTCGTTAAAAACGGTTGTCACGAAAATCGTTATGTAGATTTATTTTACTAAAACCTAATGTAACGATG
AATATCGTTATTTATATTAATCAGGGTTTGTTGGAGTTTATACAAAGTGTTCAGACTAATGATGAGTAAC
AGTGTGTTCTTCTTTGACAATATATCAATGTTTTTGTTGGGATATTGACAACTGCTACAAATAATGTCAT
GTTTTCAGGAATACTCGGTACAGTTAACGTTCCGAGAACAGTGGTTAGATGAGCGCCTCAAGTTCAAT
AATTTGGGAG

Text S4 pMIB/V5-HisB

Note: The restriction enzyme sites used in this study (Kpn I and Not I) are indicated in red font.

CATGATGATAAACAATGTATGGTGCTAATGTTGCTTCAACAACAATTCTGTTGAACTGTGTTTTCATG
TTTGCCAACAAGCACCTTTATACTCGGTGGCCTCCCCACCACCAACTTTTTTGCACTGCAAAAAAACA
CGCTTTTGCACGCGGGCCCATACATAGTACAAACTCTACGTTTCGTAGACTATTTTACATAAATAGTC
TACACCGTTGTATACGCTCCAAATACACTACCACACATTGAACCTTTTTGCAGTGCAAAAAAGTACGT
GTCGGCAGTCACGTAGGCCGGCCTTATCGGGTCGCGTCCTGTCACGTACGAATCACATTATCGGACC
GGACGAGTGTTGTCTTATCGTGACAGGACGCCAGCTTCCTGTGTTGCTAACCGCAGCCGGACGCAAC
TCCTTATCGGAACAGGACGCGCCTCCATATCAGCCGCGCGTTATCTCATGCGCGTGACCGGACACGA
GGCGCCCGTCCCGCTTATCGCGCCTATAAATACAGCCCGCAACGATCTGGTAAACACAGTTGAACAG
CATCTGTTCGAATTTAAAGCTACCATGAAATTCTTAGTCAACGTTGCCCTTGTTTTTATGGTCGTATAC
ATTTCTTACATCTATGCCGGCATGCTAAGCTTGGTACCGAGCTCGGATCCACTAGTCCAGTGTGGTGG
AATTCTGCAGATATCCAGCACAGTGGCGGCCGCTCGAGTCTAGAGGGCCCGCGGTTCGAAGGTAAGC
CTATCCCTAACCCTCTCCTCGGTCTCGATTCTACGCGTACCGGTCATCATCACCATCACCATTGAGTTT
ATCTGACTAAATCTTAGTTTGTATTGTCATGTTTTAATACAATATGTTATGTTTAAATATGTTTTTAAT
AAATTTTATAAAATAATTTCAACTTTTATTGTAACAACATTGTCCATTTACACACTCCTTTCAAGCGC
GTGGGATCGATGCTCACTCAAAGGCGGTAATACGGTTATCCACAGAATCAGGGGATAACGCAGGAA
AGAACATGTGAGCAAAAGGCCAGCAAAAGGCCAGGAACCGTAAAAAGGCCGCGTTGCTGGCGTTTT
TCCATAGGCTCCGCCCCCCTGACGAGCATCACAAAAATCGACGCTCAAGTCAGAGGTGGCGAAACCC
GACAGGACTATAAAGATACCAGGCGTTTCCCCCTGGAAGCTCCCTCGTGCGCTCTCCTGTTCCGACCC
TGCCGCTTACCGGATACCTGTCCGCCTTTCTCCCTTCGGGAAGCGTGGCGCTTTCTCATAGCTCACGC
TGTAGGTATCTCAGTTCGGTGTAGGTCGTTCGCTCCAAGCTGGGCTGTGTGCACGAACCCCCCGTTCA
GCCCGACCGCTGCGCCTTATCCGGTAACTATCGTCTTGAGTCCAACCCGGTAAGACACGACTTATCGC
CACTGGCAGCAGCCACTGGTAACAGGATTAGCAGAGCGAGGTATGTAGGCGGTGCTACAGAGTTCTT
GAAGTGGTGGCCTAACTACGGCTACACTAGAAGAACAGTATTTGGTATCTGCGCTCTGCTGAAGCCA
GTTACCTTCGGAAAAAGAGTTGGTAGCTCTTGATCCGGCAAACAAACCACCGCTGGTAGCGGTGGTT
TTTTTGTTTGCAAGCAGCAGATTACGCGCAGAAAAAAAGGATCTCAAGAAGATCCTTTGATCTTTTCT
ACGGGGTCTGACGCTCAGTGGAACGAAAACTCACGTTAAGGGATTTTGGTCATGCGAAACACGCACG
GCGCGCGCACGCAGCTTAGCACAAACGCGTCGTTGCACGCGCCCACCGCTAACCGCAGGCCAATCGG
TCGGCCGGCCTCATATCCGCTCACCAGCCGCGTCCTATCGGGCGCGGCTTCCGCGCCCATTTTGAATA
AATAAACGATAACGCCGTTGGTGGCGTGAGGCATGTAAAAGGTTACATCATTATCTTGTTCGCCATC
CGGTTGGTATAAATAGACGTTCATGTTGGTTTTTGTTTCAGTTGCAAGTTGGCTGCGGCGCGCGCAGC
ACCTTTGCCGGGATCTGCCGGGCTGCAGCACGTGTTGACAATTAATCATCGGCATAGTATATCGGCA
TAGTATAATACGACAAGGTGAGGAACTAAACCATGGCCAAGCCTTTGTCTCAAGAAGAATCCACCCT
CATTGAAAGAGCAACGGCTACAATCAACAGCATCCCCATCTCTGAAGACTACAGCGTCGCCAGCGCA
GCTCTCTCTAGCGACGGCCGCATCTTCACTGGTGTCAATGTATATCATTTTACTGGGGGACCTTGCGC
AGAACTCGTGGTGCTGGGCACTGCTGCTGCTGCGGCAGCTGGCAACCTGACTTGTATCGTCGCGATC
GGAAATGAGAACAGGGGCATCTTGAGCCCCTGCGGACGGTGCCGACAGGTTCTTCTCGATCTGCATC
CTGGGATCAAAGCCATAGTGAAGGACAGTGATGGACAGCCGACGGCAGTTGGGATTCGTGAATTGC
TGCCCTCTGGTTATGTGTGGGAGGGCTAAGCACTTCGTGGCCGAGGAGCAGGACTGACACGTCCCGG
GAGATCTGCATGTCTACTAAACTCACAAATTAGAGCTTCAATTTAATTATATCAGTTATTACCCATTG
AAAAAGGAAGAGTATGAGTATTCAACATTTCCGTGTCGCCCTTATTCCCTTTTTTGCGGCATTTTGCC
TTCCTGTTTTTGCTCACCCAGAAACGCTGGTGAAAGTAAAAGATGCTGAAGATCAGTTGGGTGCACG

AGTGGGTTACATCGAACTGGATCTCAACAGCGGTAAGATCCTTGAGAGTTTTCGCCCCGAAGAACGT
TTTCCAATGATGAGCACTTTTAAAGTTCTGCTATGTGGCGCGGTATTATCCCGTATTGACGCCGGGCA
AGAGCAACTCGGTCGCCGCATACACTATTCTCAGAATGACTTGGTTGAGTACTCACCAGTCACAGAA
AAGCATCTTACGGATGGCATGACAGTAAGAGAATTATGCAGTGCTGCCATAACCATGAGTGATAACA
CTGCGGCCAACTTACTTCTGACAACGATCGGAGGACCGAAGGAGCTAACCGCTTTTTTGCACAACAT
GGGGGATCATGTAACTCGCCTTGATCGTTGGGAACCGGAGCTGAATGAAGCCATACCAAACGACGA
GCGTGACACCACGATGCCTGTAGCAATGGCAACAACGTTGCGCAAACTATTAACTGGCGAACTACTT
ACTCTAGCTTCCCGGCAACAATTAATAGACTGGATGGAGGCGGATAAAGTTGCAGGACCACTTCTGC
GCTCGGCCCTTCCGGCTGGCTGGTTTATTGCTGATAAATCTGGAGCCGGTGAGCGTGGGTCTCGCGGT
ATCATTGCAGCACTGGGGCCAGATGGTAAGCCCTCCCGTATCGTAGTTATCTACACGACGGGGAGTC
AGGCAACTATGGATGAACGAAATAGACAGATCGCTGAGATAGGTGCCTCACTGATTAAGCATTGGTA
ACTGTCAGACCAAGTTTACTCATATATACTTTAGATTGATTTAAAACTTCATTTTTAATTTAAAAGGA
TCTAGGTGAAGATCCTTTTTGATAATCT
